# A simple and reliable protocol for mouse serum proteome profiling studies by use of two-dimensional electrophoresis and MALDI TOF/TOF mass spectrometry

**DOI:** 10.1186/1477-5956-6-25

**Published:** 2008-09-12

**Authors:** Maria Stella Ritorto, Jürgen Borlak

**Affiliations:** 1 Department of Drug Research and Medical Biotechnology, Fraunhofer Institute of Toxicology and Experimental Medicine, Nikolai-Fuchs-Str 1, 30625, Hanover, Germany; 2 Centre for Pharmacology and Toxicology, Hanover Medical School, Carl-Neuberg-Str 1, 30625, Hanover, Germany

## Abstract

**Background:**

Unravelling the serum proteome is the subject of intensified research. In this regard, two-dimensional electrophoresis coupled with MALDI MS analysis is still one of the most commonly used method. Despite some improvements, there is the need for better protocols to enable comprehensive identification of serum proteins.

Here we report a combination of two proteomic strategies, zoom in acidic and neutral part of 2-D gels and an application of two optimised matrix preparations for MALDI-MS analyses to simplify serum proteome mapping.

**Results:**

Mouse serum proteins were separated by 2-D electrophoresis at the pH ranges 3–10 and 4–7, respectively. Then in gel tryptic digests were analysed by MALDI-MS. Notably, sample-matrix preparations consisted of either a thin-layer α-ciano-4-hydroxycinnamic acid (CHCA) matrix deposition or a matrix-layer 2,5-dihydroxybenzoic acid (DHB). This enabled an identification of 90 proteins. The herein reported method enhanced identification of proteins by 32% when compared with previously published studies of mouse serum proteins, using the same approaches. Furthermore, experimental improvements of matrix preparations enabled automatic identification of mouse proteins, even when one of the two matrices failed.

**Conclusion:**

We report a simple and reliable protocol for serum proteome analysis that combines an optimized resolution of 2-D gels spots and improved sample-matrix preparations for MALDI-MS analysis. The protocol allowed automated data acquisition for both CHCA and DHB and simplified the MS data acquisition therefore avoiding time-consuming procedures. The simplicity and reliability of the developed protocol may be applied universally.

## Background

From a disease diagnostic and drug monitoring point of view there is great interest in serum proteome mapping of humans and of laboratory animals. Indeed, various mouse strains and genetically engineered animals are considered to be good models for human diseases as they offer unprecedented opportunities for mechanistic studies with new experimental medicines. There is hope that serum proteomics enables an identification of biomarkers of disease and drug safety and serum proteins can be used for therapeutic monitoring. In the past 2-D maps for human serum have been reported [1-4]. And very recently a map for the C57BL6 mouse serum proteome was published [5].

In general, serum proteome profiling is challenging, because of interference by high-abundance proteins such as albumin, immunoglobulins, antitrypsin and transferrin, which typically constitute greater than 90% of total protein mass [1,2,6-9]. These abundant proteins may hinder the detection of low-abundance proteins that can be of specific interest in the search of biomarkers of disease. Additionally, protein biochips have been applied to proteomic studies with antibody microarrays offering new possibilities in the simultaneous identification of analytes from complex samples [10].

So far, only a handful of plasma proteins are routinely measured for diagnostic purposes, because an effective technology that rapidly detects and quantifies specific changes of proteins including low-abundance proteins of serum is not available. As summarized elsewhere [11], the most common methods for serum proteome studies include separation of proteins by gel electrophoresis, excision of spots from the gel, enzymatic digestion and analysis by mass spectrometry. In particular, pre-fractionation techniques such as serum albumin depletion are useful procedures in proteome profiling studies, but they may introduce bias as well. There is substantial run-to-run variation after albumin depletion with IgY immunoaffinity spin columns. Likewise, pre-fractionation increases the risk of depletion of low-abundance proteins as has been shown for paraneoplastic antigen MA I, coagulation factor VII precursor, prostate-specific antigen, as a result of multiple protein-protein interactions with IgG, transferrins, and/or gelsolin [11,12].

In this regard, MALDI-MS is considered to be one of the most powerful techniques for the analysis of proteins and peptides [13-15], but the sample matrix preparation greatly influences the quality of MALDI-MS spectra of peptides and therefore protein identification. Despite considerable knowledge in the use of MALDI-MS [15-18], sample-matrix preparations are basically empirical.

Here we report a protocol for serum proteome profiling based on zoom in gels in the acidic and neutral pH that enabled detection of many serum proteins. Moreover, the developed protocol allowed for an automated data acquisition, and the sample protocol was optimised by the use of two different matrix-sample preparations in sequence [19]. We thus applied tryptic in-gel digest matrix preparation to either α-ciano-4-hydroxycinnamic acid (CHCA) or 2, 5-dihydroxybenzoic acid (DHB) as recently reported by us [19].

## Materials and methods

### Serum sample preparation

C57/BL6 mice (healthy mice) were obtained from Harlan Winkelman (Borchen, Germany) and kept in an animal house with 12 hour of light and dark cycled. Food and water was given *ad libitum*. Blood serum was collected from *vena cava *and allowed to clot for 2 hours at room temperature. The clotted material was removed by centrifugation at 3000 rpm for 15 min. Hemolytic material was not observed. The sera obtained from the blood samples were frozen immediately without any further treatment in liquid nitrogen and stored at -80°C until further analysis. The protein concentration of serum was determined with the Bradford protein assay (Bio-Rad Protein Assay Dye Reagent Concentrate, Bio-Rad), using bovine gamma globulin as the standard. The protein concentration ranged from 80 to 90 μg/μl for wild type mouse serum samples.

### Materials

IPG strips of pH 3 to 10 and 4 to 7 (ReadyStrip, 0.5 × 3 × 170 mm; BioRad, Germany), Bio-Lyte (pH 3 to 10), SDS, acrylamide, methylenebisacrylamide, TEMED, ammonium persulfate, DTT, urea, Tris, glycine, glycerol, and CHAPS were purchased from Bio-Rad. *Alpha-cyano-4-hydroxycinnamic acid *(CHCA) and *2,5 dihydroxybenzoic acid *(DHB) from Bruker Daltonics. Methanol, ethanol, phosphoric acid, acetic acid, and formaldehyde were purchased from Merck (Darmstadt, Germany). Sequencing grade modified trypsin was obtained from Promega (Madison, WI, USA).

Coomassie Brilliant Blue G-250, TCA, iodoacetamide and other reagents were obtained from Sigma (St. Louis, MO, USA).

### Two-dimensional electrophoresis

IEF was carried out using commercially available, dedicated apparatuses: IPGphor Protean IEF Cell (Bio-Rad). IPG strips were used according to manufacturer instructions [see also [20]]. About 500 μg of serum for gel were diluted to 350 μL with re-hydration solution (5 M urea, 2 M Thiourea, 2% CHAPS, 100 mM DTT, 0.5% v/v pH 3 to 10 IPG buffer, 40 mM Tris Base, 2% SB 3–10 and trace bromophenol blue), and applied to immobilized pH 3 to 10 nonlinear and pH 4 to 7 linear gradient strips by overnight re-hydration at 50 V. With Protean IEF Cell, focusing was done initially at 250 V for 15 min, then the voltage was increased to 10 000 V within 3 h, and maintained at 10 000 V for 7 h for a total of 70 kVh. All IEF steps were carried out at 20°C. After the first-dimensional IEF, IPG gel strips were placed in an equilibration solution (6 M urea, 2% SDS, 30% glycerol, 50 mM Tris-HCl, pH 8.8) containing 1% DTT for 10 min with shaking at 50 rpm on an orbital shaker. The gels were then transferred to the equilibration solution containing 2.5% iodoacetamide and shaken for a further 10 min before placing them on 12% polyacrylamide gel slab (185 × 200 × 1.0 mm).

Separation in the second dimension was carried out using Protean II electrophoresis equipment and Tris-glycine buffer (25 mM Tris, 192 mM glycine) containing 0.1% SDS, at a current setting of 5 mA/gel for the initial 1 h and 10 mA/gel thereafter. The second-dimensional SDS-PAGE was developed until the bromophenol blue dye marker had reached the bottom of the gel.

### Protein visualization and image analysis

Following second-dimensional SDS-PAGE, analytical gels were fixed three times in 30% ethanol containing 2% phosphoric acid for 20 min and rinsed three times in 2% phosphoric acid. Gels were then equilibrated in a solution containing 18% ethanol, 2% phosphoric acid, and 15% ammonium sulfate for 30 min and Coomassie Brilliant Blue G-250 was added to a final concentration of 1%. Staining was carried out overnight. Protein patterns in the gels were recorded as digitalized images using a high-resolution scanner (Pharox FX, Bio-Rad). Gel image matching was done with PDQuest software (Version 8.01.40; Bio-Rad). Scanned gel images were processed to remove backgrounds, staining on the gel borders and to automatically detect spots. For all spot intensity calculations, normalized values were used. Normalization of spot intensity was done with Loess Regression Method and normalized spot intensities were expressed in ppm.

### In-gel digestion

In-gel digestion of protein spots on Coomassie gels was carried out with 160 ng of Porcine Modified Trypsin (Sigma) in 10% ACN and 25 mM NH_4_HCO_3 _and performed essentially as described below.

Briefly, after the completion of staining, the gel were washed twice with water for 15 min, and then twice with water/ACN (1:1 v/v) for 15 min. The solvent volumes were about twice the gel volume. Liquid was removed, ACN was added to the gel pieces and the mixture was left for 5 min. Liquid was removed and the gel pieces were re-hydrated in 0.1 M NH_4_HCO_3 _for 5 min. ACN was added to give a 1:1 v/v mixture of 0.1 M NH_4_HCO_3_/ACN and the mixture was incubated for 15 min. All liquid was removed the digestion buffer containing 25 mM NH_4_HCO_3 _and 10 ng/μL of trypsin was added and all was incubated for 4 hours at 37°C. The supernatant was recovered and the extraction was carried out with 1% TFA/ACN (1:1 v/v).

### MALDI-TOF-MS, MS/MS and database search

Tryptic peptides were spotted directly onto a 600 μm/384 well AnchorChip™ sample target (Bruker Daltonics). The matrix CHCA was saturated in 97% Acetone/0.1% TFA solution; DHB matrix was solved in 30% ACN/0.1% TFA solution (5 mg/ml). The matrix-analyte preparations were loaded onto the MALDI plate (AnchorChip™ 600 mm 384 well, Bruker Diagnostic) by the thin layer and the matrix layer (ML) method for CHCA and DHB respectively. A further re-crystallization approach was tested on peptide calibration standard preparation (Bruker Daltonics) and applied for the samples on the AnchorChip™ [19]. An external peptide calibration standard containing the following fragments was used to calibrate the instrument: angiotensin II ([M+H]+ 1046.54); angiotensin I ([M+H]+ 1296.680); substance P ([M+H]+ 1347.740); bombesin ([M+H]+ 1619.820); ACTH clip 1–17 ([M+H]+ 2093.090); ACTH clip 18–39 ([M+H]+ 2465.200); somatostatin 28 ([M+H]+ 1347.470) (Bruker Daltonics). Furthermore, the spectra were calibrated using trypsin autolysis products (*m/z*_*s *_1045.564, 2211.108 and 2225.119) for three points internal calibration resulting in a mass accuracy of <50 ppm.

The MALDI mass spectra were obtained using an Ultraflex II TOF/TOF mass spectrometer equipped with a 384-sample scout source (Bruker Daltonics) and AutoXecute^® ^software for automatic spectra acquisition (Bruker Daltonics) was used. Peptide masses were searched against SwissProt database employing Mascot (in-house MASCOT-server) for protein identification. Database searches were performed taking into account carbamidomethyl modification of cysteines and possible oxidation of methionine and allowing one missed cleavage. The mass accuracy required for PMF and MS/MS was basically chosen according to peptide mass-tolerance defined by Root Mean Square (RMS) error, since it defines the limit of peptide mass tolerance (*Peptide tol +-*) for respectively Mascot Peptide Mass Fingerprint and Mascot MS/MS Ion Search to obtain a significant score (p < 0.05) of matched peptides to select protein entry [21].

Peptides were identified using ProteinScape™ database (Protagen, Bruker, Germany) and Mascot search engine to cross-validate or consolidate the identification results through the complementary use of several software packages. We use ProteinScape™ ScoreBooster feature to improve database search results by automatic iterative recalibrations and background eliminations. Identified proteins were checked individually for further considerations.

The criteria used to accept identifications included the extent of sequence coverage, the number of matched peptides, the Mascot score, i.e. the *Top Score *obtained after Mascot has incorporated the Mowse score into a probabilistic framework. It is defined as -10*LOG_10_(P), where P is the absolute probability that the observed match is a random event [21]. In case of SwissProt as selected protein sequence database, protein scores greater than 53 were significant (p < 0.05). In addition we requested mouse protein as the top candidates in the first pass search when no restriction was applied to the species of origin. Moreover, when the identification was not possible to reach from PMF, MS/MS data were collected and used as result, if *Significant hits *were obtained by Probability Based Mowse Score (individual ion scores >25 that indicate identity or extensive homology-p < 0.05-) [21]. In general, false positive evaluation was done by software with randomized searches to remove redundant peptide and protein identification.

The new identifications were furthermore searched in PeptideMap free software (ProWL free software programs, National Resource for the Mass Spectrometric Analysis of Biological Macromolecules, the Rockefeller University) [22] and compared with Mascot results (for details, please refer to Additional files 1, 2).

## Results and discussion

### Two-dimensional electrophoresis of serum proteins

**Figure 1:**
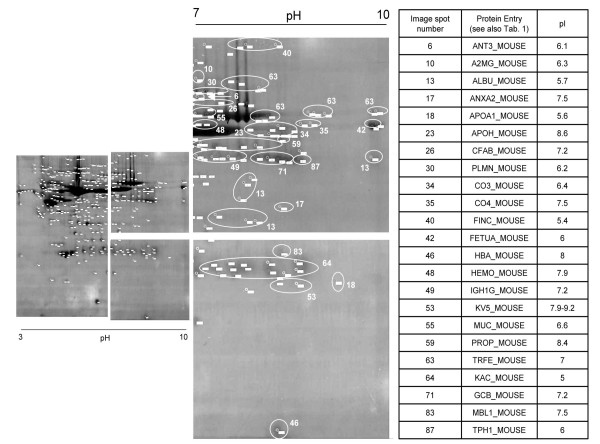
**Protein entries at basic region of 3–10 pH range**. An amount of 23 proteins were identified at basic region of gels at pH range 3–10. As discussed in the text, most of those proteins could be relevant in biomarker discovery research because of their involvement in inflammation or in mechanisms that could bring toward the development of cancer.

We found narrow 2-D gels to improve detection of low abundant proteins in sera, thereby avoiding any pre-fractionation. The 2-DE proteomics carries the advantage of visualizing changes in Mw and pI of a protein, which we find helpful in highlighting biologically significant processes. This electrophoresis technique has been applied successfully to identify oncoproteins in human serum and tissues [11,13,20,23]. Notably, mouse serum proteins were separated by 2-D electrophoresis (2-DE) and resolved in the first dimension in a broad pH range with IPGphor strips (pH 3 to 10 NL), and subsequently in a 12% gradient polyacrylamide SDS gel in the second dimension. On average, 350 spots could be detected and 77 unique mouse proteins were identified, of which more than 30% were in the basic region of the gel (Figure 1). Among them, we identified some proteins already known or evaluated as potential biomarkers for human malignancies. For instance, previous studies have shown the benefit of studying complement factors in human cancer, i.e. elevated CO3 levels identified in pancreatic cancer patients. Consequently, monitoring activity of CO3/CO4 is clinically relevant [24-26]. Moreover, heme in tissues and circulation may be come toxic due to oxygen radical formation. Hemopexin, a plasma protein binds heme with high affinity, and therefore limits its reactivity thereby facilitating its catabolism via receptor-mediated endocytosis. Furthermore, the heme-hemopexin complex is glycosilated at specific Hys residues. Obviously, monitoring the precursor at basic pH and its glycosilated form at acidic pH can be informative as biomarker of disease [27,28]. Likewise, we identified tryptophan 5-hydroxylase 1 (TPH1_MOUSE) in the serum proteome. This protein promotes the synthesis of 5-HT (serotonin). Intriguingly, a recent investigation has found its involvement in the regulation of the immunosystem and aberrant activity in cancers [29,30].

**Figure 2:**
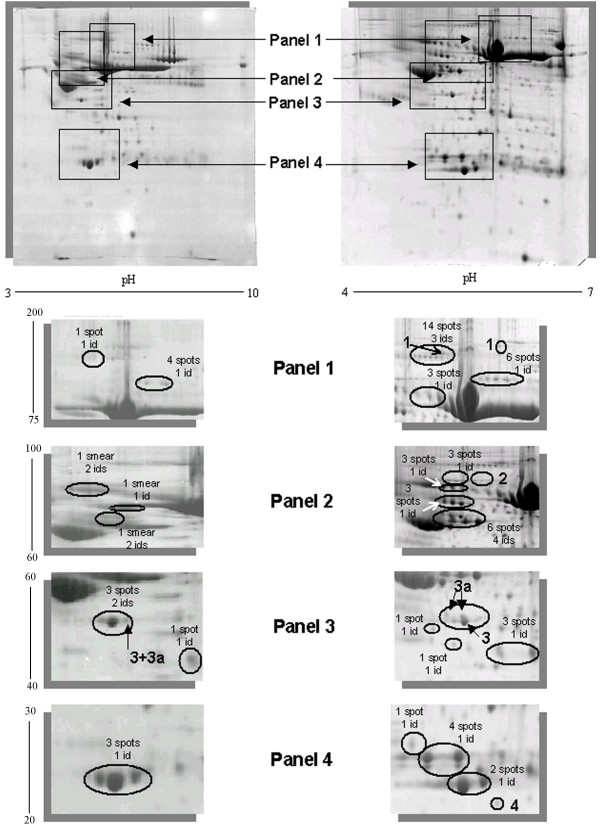
**Improved resolution with zoom-in 2-D gels**. Details of the 2-D gel zoomed areas. We showed the improved separation and visualization of the mouse serum proteome. In fact, multiple isoforms for most identified proteins were found and other identified spots were detected only in 4–7 pH-range and not in 3–10 pH-range. Panel 1: *3–10 pH range *(1 spot 1 id: *mouse ceruloplasmin; *4 spots 1 id: *mouse gelsolin*) *4–7 pH range *(14 spots 3 ids: *mouse ceruloplasmin, mouse alpha-macroglobulin, mouse albumin*; 6 spots 1 id: *mouse gelsolin*; 3 spots 1 id: *mouse hemopexin*).Panel 2: *3–10 pH range *(1 smear 2 ids: *mouse afamin *and *mouse hemopexin; *1 smear 1 id: *mouse kininogen; *1 smear 2 ids: *mouse antithrombin-III, mouse Alpha-2-HS-glycoprotein*) *4–7 pH range *(3 spots 1 id: *mouse prothrombin; *3 spots 1 Id: *mouse hemopexin; *3 spots 1 id: *mouse kininogen; *6 spots 4 ids: *mouse antithrombin-III, mouse Alpha-2-HS-glycoprotein, mouse vitamin D-binding protein and mouse fetuin-b*).Panel 3: *3–10 pH range *(3 spots 2 ids: *mouse apolipoprotein A4 and mouse zinc-alpha-2-glycoprotein; *1 spot 1 id: *mouse albumin*) *4–7 pH range *(1 spot 1 id: *mouse serum paraoxonase/arylesterase 1; *1 spot 1 id: *mouse H-2 class I histocompatibility antigen; *3 spots 1 id: *mouse alpha-2-macroglobulin)*. Panel 4: *3–10 pH range *(3 spots 1 id: *mouse apolipoprotein A1*) *4–7 pH range *(1 spot 1 id: *mouse mannose-binding protein2; *4 spots 1 id: *mouse Ig kappa chain V-III region; *2 spots 1 id: *mouse apolipoprotein A2*). The spots 1 (alpha-2-macroglobulin); 2 (complement C1r-subcomponent); 3 and 3a (Apoliprotein A4 and Zinc-alpha-glycoprotein 2 respectively) and 4 (glutathione peroxidase 3) are examples discussed respectively in the text and in Figure 5.

As shown in Figure 2 the sample complexity was further reduced by the use of IPG strips in the pH range of 4 to 7. The number of spots was increased by 2-fold. Approximately 660 spots were detected and 59 proteins were identified in this pH range. When compared with gels of the 3 to 10 pH-range, multiple isoforms of proteins can now be visualised and may have arisen from a combination of post-translational modifications as well as chemical modifications that occur during sample preparation (Figure 2) [20,31]. Apart from high abundant proteins, on average three spots for each protein at pH of 4 to 7 could be detected, when compared to one spot for each entry or smears observed with gels at pH of 3 to 10 (Figure 2). Altogether, the spot detection was improved and a total of 16 of these proteins were novel gene products resolved at pH 4 to 7 (Table 1, *italic font*). Notably, 4 of them had not been reported so far in mouse serum [5-9,32,33].

**Table 1:** Mouse serum protein map

No.	SwissProt entry	SwissProt accession	Mw (KDa)	pI	Mascot score	sequence coverage (%)	*msms (m/z)*	*matrix*	Our Map 1 (matched peptides)	Ref Map 2 (matched peptides)	Ref Map 3 (matched peptides)	Ref Map 4 (matched peptides)	Ref Map 5 (matched peptides)	Ref Map 6 (matched peptides)
**1**	A1AG1 MOUSE	Q60590	24	5.6	124	32	1703.91	CHCA DHB	10	**not present**	4	12	5 (up regulated)	present
***2***	***AMBP MOUSE***	***Q07456***	***40***	***6***	***38***	***4***	***1669.85***	***CHCA ***	***1***	3*	***not present***	***not present***	***not present***	***not present***
**3**	APOM MOUSE	Q9Z1R3	21.6	6	105	36	938.42	CHCA DHB	9	**not present**	**not present**	**not present**	**not present**	present
**4**	MUP1 MOUSE	P11588	20.9	5	115	55		DHB	12	0	10	**not present**	**not present**	**not present**
	MUP2 MOUSE	P11589	20.9	5	146	68		DHB	14	0	11	19	**not present**	**not present**
	MUP6 MOUSE	P02762	20.9	5	136	62		DHB	12	0	10	**not present**	**not present**	**not present**
	MUP8 MOUSE	P04938	17.7	5	141	74		DHB	12	0	10	**not present**	**not present**	**not present**
**5**	A1AT1 MOUSE	P07758	46.1	5.4	145	41	*981.56* *1137.66* *1232.70* *2003.04* *2327.09* *2405.17* *3498.80*	CHCA DHB	15	10* 2'	13	12	20 (up regulated)	**not present**
	A1AT2 MOUSE	P22599	46.1	5.3	104	36	*2405.17* *2003.04* *2327.09* *3498.80*	CHCA DHB	12	9'	**not present**	7	3 (up regulated)	present
	A1AT3 MOUSE	Q00896	46	5.3	146	44	*981.56* *1137.66* *2003.04* *2327.09* *2405.17* *3498.80*	CHCA DHB	15	**not present**	16	15	**not present**	**not present**
	A1AT4 MOUSE	Q00897	46.1	5.2	162	51	*1232.74* *2003.04* *2327.09* *3498.80*	CHCA DHB	17	**not present**	**not present**	**not present**	**not present**	present
	A1AT5 MOUSE	Q00898	46	5.4	105	35	*1232.71* *2327.09* *2405.17*	CHCA DHB	12	**not present**	**not present**	**not present**	**not present**	present
	A1AT6 MOUSE	P81105	46	5.2	161	47	*981.56* *1137.66* *1232.70* *2003.04* *2327.09* *2405.17* *3498.80*	CHCA DHB	16	2	**not present**	8	**not present**	present
**6**	ANT3 MOUSE	P32261	52.5	6.1	298	56	*1198.70* *1340.69* *1359.67* *1700.89*	CHCA DHB	29	6	**not present**	**not present**	**not present**	present
**7**	A2AP MOUSE	Q61247	55.1	5.8	169	36	*1591.80* *1680.81*	CHCA DHB	15	3	**not present**	**not present**	**not present**	present
**8**	CBG MOUSE	Q06770	44.9	5	148	32		CHCA DHB	10	3	**not present**	**not present**	**not present**	present
**9**	SPA3K MOUSE	P07759	47	5	113	29		CHCA DHB	12	12	**not present**	21	7 (up regulated)	present
**10**	A2MG MOUSE	Q61838	167	6.3	153	19	*2068.21*	CHCA DHB	18	25	16 (immuno depletion)	**not present**	**not present**	present
**10a**	A2MG MOUSE C-term	Q61839	*28*	*7*	62	6	*1031.50* *1111.58* *1216.61* *1787.96*	CHCA DHB	9	?	**not present**	13	16 (up regulated)	present
**11**	*ADIPO MOUSE*	*Q60994*	*26.9*	*5.3*	*63*	*19*	*1504.72*	*DHB*	*5*	***not present***	***not present***	***not present***	***not present***	*present*
**12**	AFAM MOUSE	O89020	71.5	5.5	155	31		CHCA DHB	15	1	9	**not present**	**not present**	present
**13**	ALBU MOUSE	P07724	70.7	5.7	426	68	*1455.83* *1479.70* *1609.90* *1882.96* *1960.15* *1981.98*	CHCA DHB	40	26* 46'	17	48	12 (up regulated)	present
**14**	*ALS MOUSE*	*P70389*	*67.7*	*6.1*	*97*	*28*		*DHB*	*13*	***not present***	***not present***	***not present***	***not present***	*present*
**15**	CPN2 MOUSE	Q9DBB9	61.3	5.5	131	25		CHCA	10	**not present**	**not present**	**not present**	**not present**	present
**16**	ANGL6 MOUSE	Q8R0Z6	51.4	9.2	56	16		DHB	4	**not present**	**not present**	**not present**	**not present**	**not present**
**17**	ANXA2 MOUSE	P07356	38.8	7.5	82	18		CHCA	5	**not present**	**not present**	**not present**	**not present**	**not present**
**18**	APOA1 MOUSE	Q00623	30.5	5.6	207	49	*1237.67* *1318.61* *1331.53* *1340.77*	CHCA DHB	17	5* 18'	13	18	22	present
**19**	APOA2 MOUSE	P09813	11.3	6.6	85	14	*1193.62* *1831.98*	CHCA DHB	2	4'	7	3	3 (up regulated)	present
**20**	APOA4 MOUSE	P06728	45	5.4	223	60	*1131.66* *1231.61* *1461.75* *2023.05*	CHCA DHB	21	2* 5'	15	29	6 (up regulated)	present
**21**	APOE MOUSE	P08226	35.9	5.6	166	51	*968.52* *1075.60* *1599.82*	CHCA DHB	21	5'	7	17	16 (up regulated)	present
**22**	**APOC3 MOUSE**	**P33622**	**10.9**	**4.6**	**140**	**19**	***1062.46*** ***1078.45*** ***1987.94***	**CHCA**	**3**	**not present**	3	3	**not present**	present
**23**	APOH MOUSE	Q01339	39.9	8.6	276	62	*1325.68* *1544.85* *2719.48*	CHCA DHB	22	7	**not present**	17	22 (up regulated)	present
**24**	CLUS MOUSE	Q06890	55.2	5.5	103	19		*CHCA DHB*	11	2*	9	12	**not present**	present
***25***	*C1R MOUSE*	*Q8CG16*	*81.5*	*5.4*	*78*	*17*		*CHCA*	*8*	***not present***	***not present***	***not present***	***not present***	***not present***
**26**	CFAB MOUSE	P04186	86.3	7.2	269	37		CHCA DHB	23	3	**not present**	**not present**	**not present**	present
***27***	*CFAI MOUSE*	*Q61129*	*69.5*	*7.4*	*103*	*21*	*1726.85*	*DHB*	*11*	*3*	***not present***	***not present***	***not present***	***not present***
***28***	*HGFA MOUSE*	*Q9R098*	*72.9*	*6.6*	*58*	*13*		*DHB*	*6*	***not present***	***not present***	***not present***	***not present***	*present*
**29**	HPT MOUSE	Q61646	39.2	5.9	146	49	*980.49* *1320.74* *1373.61*	DHB	21	**not present**	11	5	14-3 (up and down regulated)	present
**29a**	HPT MOUSE	Q61646			81	11	*1679.78*	DHB	6	?	?	14	?	**not present**
**30**	PLMN MOUSE	P20918	93.4	6.2	387	53	*1138.46*	CHCA DHB	35	8	**not present**	32	6	present
**31**	CFAH MOUSE	P06909	144	6.6	269	31		CHCA DHB	29	6	**not present**	18	**not present**	present
***32***	*CS1A MOUSE*	*Q8CG14*	*78.3*	*5*	*66*	*15*		*DHB*	*6*	***not present***	***not present***	***not present***	***not present***	***not present***
**33**	F13B MOUSE	Q07968	78.3	5.6	148	26		CHCA	15	**not present**	**not present**	**not present**	**not present**	present
**34**	CO3 MOUSE	P01027	188	6.4	312	29	*1886.93*	CHCA DHB	40	23* 1'	**not present**	**not present**	17 (up regulated)	present
**35**	CO4 MOUSE	P01029	194	7.5	123	9		CHCA	14	3	**not present**	**not present**	**not present**	present
**36**	CO9 MOUSE	P06683	63.2	5.6	80	29		DHB	14	1	**not present**	**not present**	**not present**	**not present**
**37**	C1QB MOUSE	P14106	27	8.6	69	20		*CHCA*	5	1*	**not present**	**not present**	**not present**	present
**38**	EGFR MOUSE	Q01279	138	6.5	171	16		*CHCA DHB*	15	**not present**	**not present**	18	**not present**	present
**39**	FIBB MOUSE	Q8K0E8	55.4	6.7	112	39		CHCA DHB	20	**not present**	**not present**	**not present**	**not present**	present
**40**	FINC MOUSE	P11276	276	5.4	90	8		*CHCA*	16	9*	**not present**	**not present**	**not present**	present
**41**	FCN1 MOUSE	O70165	36.8	6	65	12		DHB	5	**not present**	**not present**	8	**not present**	**not present**
**42**	FETUA MOUSE	P29699	38.1	6	135	47	*1653.75 2138*	CHCA DHB	11	3	6	8	**not present**	present
**43**	FETUB MOUSE	Q9QXC1	43.5	6.2	113	30	*1382.89* *1159.7*	CHCA DHB	14	1	5	13	**not present**	present
**44**	GPX3 MOUSE	P46412	25.6	8.3	98	45	*1955*	CHCA DHB	12	**not present**	**not present**	**not present**	**not present**	present
**45**	HA10 MOUSE	P01898	37.2	5.2	173	46	*1671.86*	CHCA DHB	16	3	**not present**	**not present**	**not present**	**not present**
**46**	HBA MOUSE	P01942	15	8	79	31	*1589.82* *1819.93*	CHCA	5	2* 6'	**not present**	**not present**	8	present
**47**	HBB1 MOUSE	**P02088**	**16**	**7.3**	**68**	73	***1274.72***	CHCA DHB	**9**	**not present**	**not present**	**not present**	7 (up regulated)	present
**48**	HEMO MOUSE	Q91X72	52	7.9	188	43	*1100.47* *1212.63* *1504.76* *1516.71* *1727.77* *2472.12*	CHCA DHB	21	14	8	25	3 (up regulated)	present
**49**	IGHG1 MOUSE	P01868	36.2	7.2	94	45		DHB	9	3	**not present**	**not present**	**not present**	**not present**
**50**	KNG1 MOUSE	O08677	74.1	6	164	29	*1010.56* *1060.56* *1515.68*	CHCA DHB	18	9	12	24	5 (up regulated)	**not present**
**51**	KLKB1 MOUSE	P26262	73.4	8.4	*105*	22		CHCA	14	1*	2	**not present**	**not present**	present
**52**	***KV3A1/2/4 MOUSE***	***P01654***	***12***	***5***	***116***	***16***	***1616.89*** ***1855.04***	***CHCA DHB***	***2***	***not present***	***not present***	***not present***	***not present***	***present***
	***KV3AD MOUSE***	***P01665***	***12***	***4.9***	***54***	***44***	***1155.46*** ***1616.89*** ***1855.04***	***CHCA DHB***	***4***	***not present***	***not present***	***not present***	***not present***	***present***
**53**	***KV5AB MOUSE***	***P01644***	***12***	***7.9***	***88***	***17***	***1028.56*** ***1926.92*** ***2455.35***	***CHCA DHB***	***3***	***not present***	***not present***	***not present***	***not present***	***not present***
	***KV5J MOUSE***	***P01645***	***11.9***	***9.2***	***93***	***16***	***1926.81***	***CHCA DHB***	***1***	***not present***	***not present***	***not present***	***not present***	***present***
**54**	**KV3N MOUSE**	**P01666**	**12**	**4.5**	**58**	**53**		DHB	**3**	**not present**	**not present**	**not present**	**not present**	present
**55**	MUC MOUSE	P01872	50	6.6	184	30	*1330.78* *1603.91*	CHCA DHB	14	4	**not present**	**not present**	**not present**	**not present**
**56**	MUG1 MOUSE	P28665 (Q80XE6)	166.4	6	168	20		CHCA DHB	24	12	**not present**	**not present**	**not present**	present
**57**	PHLD MOUSE	O70362	93.8	6.6	151	23		CHCA DHB	20	1	**not present**	**not present**	**not present**	present
**58**	PON1 MOUSE	P52430	34.6	5	117	32	*1853.9*	CHCA DHB	8	2	**not present**	**not present**	**not present**	present
**59**	PROP MOUSE	P11680	50	8.4	87	21		CHCA	8	**not present**	**not present**	**not present**	**not present**	present
**60**	RETBP MOUSE	Q00724	23.5	5.7	138	59	*1226.63* *1360.58* *1789.84* *2079.88*	CHCA DHB	15	1	**not present**	**not present**	**not present**	present
**61**	SAMP MOUSE	P12246	26.4	6	88	38	*2133.03*	CHCA DHB	8	1	8	8	**not present**	present
**62**	THRB MOUSE	P19221	71.6	6	156	28	*1189.56*	CHCA DHB	19	4	**not present**	26	9 (up regulated)	present
**63**	TRFE MOUSE	Q921I1	78.8	7	308	51	*1171.61* *1419.86* *1656.81* *1990.82* *2007.94*	CHCA DHB	38	39* 1'	?	26	**not present**	present
**64**	KAC MOUSE	P01837	11.9	5	91	87	*990.51*	CHCA DHB	8	1	**not present**	**not present**	**not present**	**not present**
**65**	ZA2G MOUSE	Q64726	35.4	5.8	173	55	*1274.60* *1318.81* *1409.72* *1610.74*	CHCA DHB	15	1	**not present**	19	**not present**	present
**66**	VTDB MOUSE	P21614	55.1	5.4	266	44	*1051.6* *1303.77* *2441.13*	CHCA DHB	24	3	12	31	**not present**	present
**67**	TTHY MOUSE	P07309	15.9	5.8	122	67	*1382.62* *1554.89* *2438.17* *2517.22*	CHCA DHB	8	4* 1'	10	9	**not present**	present
**68**	GELS MOUSE	P13020	86.3	5.8	233	39	*1254.75* *1275.73*	CHCA DHB	22	6	**not present**	19	**not present**	present
**69**	VTNC MOUSE	P29788	55.6	5.7	82	23		CHCA DHB	10	2	**not present**	**not present**	**not present**	present
***70***	***GCAB MOUSE***	***P01864***	***37***	***8.5***	***31.5***	***5***	***1913.84***	***DHB***	***1***	***2 (membrane form)***	***not present***	***not present***	***not present***	***not present***
**71**	GCB MOUSE	P01866	37.3	7.2	79	30	*1778.87*	CHCA DHB	8	**3 (membrane form)**	**not present**	**not present**	**not present**	**not present**
**72**	LAC1 MOUSE	P01843	11.7	5.9	70	61		DHB	4	**not present**	**not present**	**not present**	**not present**	**not present**
**73**	LAC2 MOUSE	P01844	11.4	5.9	70	89		DHB	5	**not present**	**not present**	**not present**	**not present**	present
**74**	ACTG MOUSE	P63260	42.1	5.3	188	53	*1198.75* *1790.93* *1954.14*	CHCA DHB	16	**not present**	**not present**	**not present**	**not present**	**not present**
	ACTB MOUSE	P60710	42	5.3	171	49	*1198.75* *1790.93* *1954.14*	CHCA DHB	15	**not present**	**not present**	**not present**	**not present**	present
**75**	APC MOUSE	Q61315	313	7.4	53	6		CHCA	15	**not present**	**not present**	**not present**	**not present**	present
***76***	*CT160 MOUSE*	*Q8VCC6*	*22*	*9.5*	*56*	*20*		*CHCA*	*5*	**not present**	**not present**	**not present**	**not present**	**not present**
***77***	*CUL1 MOUSE*	*Q9WTX6*	*90.3*	*8.2*	*62*	*15*		*CHCA*	*7*	***not present***	***not present***	***not present***	***not present***	***not present***
**78**	ESTN MOUSE	P23953	61.4	5.1	168	37	*911.45*	CHCA DHB	21	12	7	**not present**	**not present**	present
**79**	ITIH2 MOUSE	Q61703	106	6.8	150	24	*1337.73*	CHCA DHB	14	2	**not present**	**not present**	**not present**	present
**80**	ITIH4 MOUSE	9055252	104.8	6	133	20		CHCA	17	**not present**	**not present**	18	**not present**	**not present**
**81**	K2C5 MOUSE	Q922U2	62	7.6	60	16		DHB	11	**not present**	**not present**	**not present**	**not present**	**not present**
**82**	LIFR MOUSE	P42703	123.8	5.7	151	19		DHB	15	**not present**	**not present**	22	**not present**	present
**83**	MBL1 MOUSE	P39039	25.8	7.5	100	27	*1544.89*	CHCA	9	**not present**	**not present**	**not present**	8 (up regulated)	present
**84**	MBL2 MOUSE	P41317	26.3	5	70	29	*1323.60* *1522.72* *1766.83*	CHCA DHB	6	2	**not present**	8	**not present**	**not present**
**85**	OST5 MOUSE	Q8BSL4	40.7	9.7	70	23		CHCA	6	**not present**	**not present**	**not present**	**not present**	**not present**
**86**	SYT2 MOUSE	P46097	47.7	8.2	61	19		CHCA	6	**not present**	**not present**	**not present**	**not present**	present
***87***	*TPH1 MOUSE*	*P17532*	*51.9*	*6*	*59*	*17*		*CHCA*	*7*	***not present***	***not present***	***not present***	***not present***	***not present***
***88***	*KPYM MOUSE*	*P52480*	*58*	*7*	*53*	*30*		*DHB*	*12*	***not present***	***not present***	***not present***	***not present***	***not present***
***89***	*DYN2 MOUSE*	*P39054*	*98*	*7*	*68*	*12*		*CHCA DHB*	*9*	***not present***	***not present***	***not present***	***not present***	***not present***
***90***	***GAB2 MOUSE ***	***Q9Z1S8***	***73.6***	***8.5***	***33***	***2***	***1794.8***	***DHB***	***1***	***not present***	***not present***	***not present***	***not present***	***present***

Mouse serum proteins identified by us in serum from C57BL6 mice (Our Mapping 1), in comparison with previous mouse serum maps (Ref Map 2: [9], Ref Map 3: [8], Ref Map 4: [7,32], Ref Map 5: ref [33] and Ref Map 6: [6]). Exactly, 16 of them (*italic font*) were identified only by narrow IPGs of pH 4–7. **In bold**, we highlighted the proteins identified only with LIFT-MS/MS measurements. In case of TCA/methanol precipitation method (Ref Map 2: [9]) we specified where the proteins were identified. (n*: precipitated fraction; n': supernatant fraction-). In the case of MUPs, we marked with 0 the entries, because there are evidences that those proteins are present just in C57BL6 mice and not in BALA/cj inbred strain [32].

### Improved MALDI TOF-MS analysis of serum proteins

**Figure 3:**
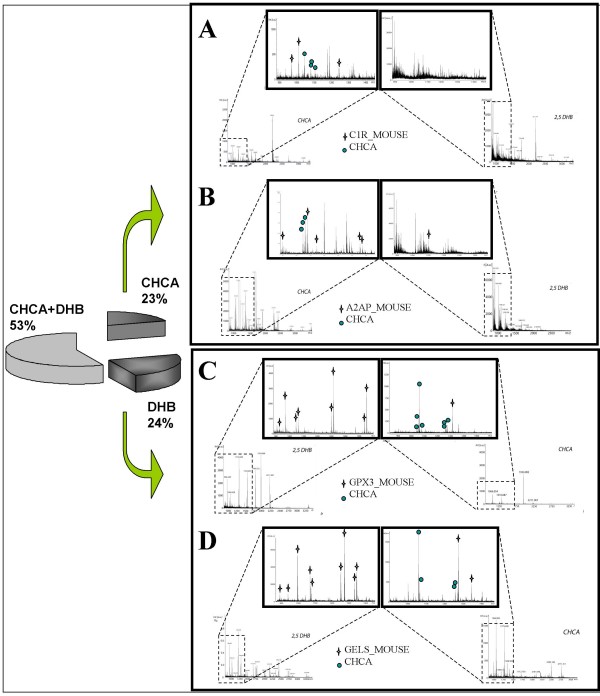
**Spectra comparisons between CHCA and DHB**. (A, B) CHCA matrix vs DHB matrix. Considerably, in the case of DHB the peptide ions signals are less resolved than other signals in the spectrum, maybe connected to metastable decay of ions in the drift tube or "chemical noises" from matrix ions. On the other hand, CHCA was enabled to identify *complement C1r-subcomponent *(C1r_MOUSE) and *alpha-2-antiplasmin *(A2AP_MOUSE). Crosses represent matched peptides to the identification. (C, D) DHB matrix vs CHCA matrix. The spectra from DHB are notably rich of peptides ions fragments (crosses) which belong to the identification, i.e. *glutatione peroxidase-secreted form *(GPX3_MOUSE) and *gelsolin *(GELS_MOUSE). The blue circles on CHCA spectra, instead, represent matrix fragments which hide the peaks could be matched to the identifications. The pie chart represents our mouse proteome mapping, where both matrices have the almost same input in the identifications.

To increase the number of ionized tryptic-digested peptides, we took advantage of the properties of two commonly used matrices, CHCA and DHB [15,19]. To the best of our knowledge sample-matrix preparations with both matrices in sequence for serum proteome profiling has not been reported so far. These matrices differ considerably upon MALDI ionization. We combined a thin layer and the ML deposition on the AnchorChip™ to identify 2-D spots from mouse serum samples using a protocol recently described by us [19]. Briefly, we used a Bruker MALDI target plate, equipped with hydrophilic patches ("anchors") in hydrophobic surroundings (AnchorChip™, Bruker Daltonics), and achieved signal uniformity, high sensitivity and an improved signal-to-noise ratio which gave rise to less complex MS spectra of which an example is given in Figure 2. Moreover, the re-crystallization of the sample-matrix mixtures loaded on the target increased considerably the number of identifiable peptide ions in an automated MS and MS/MS spectra acquisition mode (see also Table 1). Striking differences in the MALDI MS spectra of peptides were observed when the two matrices were compared (Figure 3). We observed better ionization of peptides from the tryptic-digested proteins with DHB as compared with CHCA. Indeed with CHCA, we frequently observed peaks at m/z 1044, 1060, 1066, 1082, 1249 and 1271 (Figure 3, azure cycles). We used matrix ion suppression in deflection mode of m/z 800. These abundant matrix clusters display high s/n ratio and resulted in poor acquired PMF spectra [11,34]. Previous reports had already highlighted differences in PMF because of different behaviour of the two matrices [15,16,18]. In particular, Zhu and Papayannopoulos tested several matrices and reported that DHB gave the best results without interferences from matrix ion peaks. Furthermore, common interferences from matrix-adducts of CHCA were observed, in particular in the range of m/z 800–1100 of the MALDI spectra [35].

Nonetheless, there are obvious benefits in the use of the CHCA matrix in the MALDI MS analysis. This included a high uniform layer, which it forms, especially on the AnchorChip™ (data not shown). Its ability to induce desorption of ions at lower laser energies demonstrates transfer of sufficient energy for the pre-formation of ions. In some cases, metastable ions of matrix ion fragments were less formed when compared with DHB [36]. This enabled acquisition of less ambiguous spectra for the identification of proteins. The different behaviours of both matrices are the subject of several published studies. In particular, Luo et al. [37] reported loss of internal energy of ions generated by MALDI and the role of the two matrices in desorption and ionisation processes.

Indeed, in a complex mixture such as serum, it is not really clear why in some cases diagnostic PMFs are obtained with DHB rather than CHCA and vice versa. Some examples, depicted in Figure 3, clearly demonstrate that it is difficult to predict which matrix would deliver best PMFs. For that reason, we exploited both matrices in order to redress any drawbacks of one matrix by the use of the other one especially in automatic data acquisition procedures.

### Mapping of the mouse serum

As shown in Table 1, we were able to identify 90 unique proteins, some of which were reported to be identifiable only after sample pre-treatment or sophisticated and time-consuming procedures. For instance, several members of complement factor family [CFAB_MOUSE, CFAI_MOUSE, CO3_MOUSE, CO4_MOUSE, CO9_MOUSE and C1QB_MOUSE], properdin [PROP_MOUSE] and mannose binding protein A [MBL1_MOUSE] were identified at the basic pH region of the 2-D gels. None of them were reported in previous mouse serum proteome profiling studies using 2-DE and MS analysis [7,8,32]. Indeed, when the serum was albumin-depleted, other high abundance proteins were lost as well. Furthermore, complement factor 3 and transthyretin were found in both fractions, albumin-rich and depleted (Table 1) [9]. This necessitated repetitive analyses. Indeed, complement factor 3 was shown to be up-regulated in human lung adenocarcinomas [33], as were increased serum levels of transthyretin and down-regulation of transferrin in human type-2 diabetes [38].

The application of our protocol enabled an identification of properdin [PROP_MOUSE] and adenomatous polyposis coli protein [APC_MOUSE]. Once again, in previous mouse serum maps these proteins were not identified [7-9,32,33] (Table 1). We were able to identify most of the proteins but some new proteins were identified as well (Table 1). Notably, with our optimized MALDI- matrix protocols, we were able to characterize the protein C1R complement [C1R_MOUSE] by the sequential use of DHB and CHCA (**spot 2 **in Figure 2, Figure 3). This is a serine protease that combines with C1q and C1s (which we had identified in our sample as well) to form C1, the first component of the classical pathway of the complement system. The spot corresponding to C1R is positional in the gel where the molecular weight and pH differs from the theoretical values, presumably because of glycosylation of this protein [39]. Post-translational modifications (PTMs) do change the chemical characteristics of the protein that runs in 2-D gels at more acidic and higher mass ranges. Indeed, we identified glutathione peroxidase 3 [GPX3_MOUSE] (pI 8.3) in 2-D gel at pH 4–7 (**spot 4 **in Figure 2, Figure 3). The gene product GPX3_MOUSE is a secreted protein that protects cells and enzymes from oxidative damage, by catalyzing the reduction of hydrogen peroxide, lipid peroxides and organic hydroperoxide. This protein was reported to be regulated in various malignancies [40].

**Figure 4:**
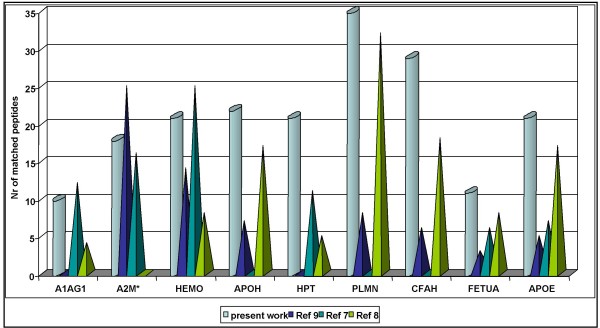
**Comparison of matched peptides**. We have depicted here a comparison of some identification (x-axis) from our work (azure-cylinder) and three different mouse serum maps (prisms) [see ref [9,7,8]]. Note the number of matched peptides (y-axis) is higher or comparable with the pre-fractionation methods. Mouse protein identifications: A1AG1: alpha-1-acid glycoprotein, A2M: alpha-2-macroglobulin, HEMO: hemopexin, APOH: beta-2-glycoprotein 1, HPT: haptoglobin, PLMN: plasminogen, CFAH: complement factor H, FETUA: alpha-2-HS-glycoprotein-Fetuin-A, APOE: apoliproteinE.

Without sample pre-treatments, we were able to identify proteins such as hemopexin [HEMO_MOUSE] and alpha-2-macroglobulin [A2MG_MOUSE]. Usually, an identification and quantification of these proteins requires time consuming procedures [9,32]. Here we describe a simple protocol that allowed facile identification of the same proteins even with a higher number of matching peptides. For instance, **spot 1 **(see panel 1 in Figure 2) was identified as A2MG_MOUSE with high sequence coverage. Previously, pre-fractionation of the serum was needed to enable its identification [32]. Likewise, with our protocol HEMO_MOUSE was identified with a Mascot score of 188 and 21 matched peptides, a result comparable to after immunodepletion (25 matched peptides), and pre-fractionation (14 matched peptides) but considerable better than with data from other mouse serum maps (8 matched peptides) [7-9]. In Figure 4, we display identified proteins where the number of matched peptides amongst different strategies was compared; in general, we obtain a better protein score and comparable or even higher number of matched peptides of the protein sequence, without any sample pre-treatment (cylinders in Figure 4, Table 1).

When no significant PMF entries were obtained, we attempted identification by MS/MS spectra acquisition. For instance, we acquired the LIFT TOF-TOF spectrum of the peak yielded at m/z 1913.84 -the peptide sequence APQVYVLPPPAEEMTKK- with an ion score of 33 and error tolerance of 10 ppm. The peptide was then matched to Ig gamma-2A chain C [GCAB_MOUSE]. Additionally, we identified the secreted protein alpha-1-microglobulin (AMBP_MOUSE) by MS/MS with the parental ion at m/z 1669.85 and a peptide sequence of TIAACNLPIVQGPCR. We thus obtained the *significant hit *at ion score of 38 with 5 ppm as peptide mass tolerance; according to RMS error, and 0.5 Da as fragment mass tolerance [21].

Furthermore, we identified a number of proteins such as gamma actin [ACTG_MOUSE] which was believed to be primarily cytoskeletal and/or intracellular but was not reported so far for the mouse serum proteome. Three peaks belonging to ACTG_MOUSE were identified at m/z 1198.75 -peptide sequence AVFPSIVGRPR- 1790.93 -peptide sequence SYELPDGQVITIGNER- and 1954.14 -peptide sequence VAPEEHPVLLTEAPLNPK-, with an ion scores higher than 23. Further examples are listed in Table 1 (from number 74 to 90).

Taken collectively, our protocol enabled automated data acquisition and improved significantly the identification of proteins based on higher sequence coverage and the number of matched peptides. For instance, CFAH_MOUSE was identified with a Mascot score of 269, a 31% sequence coverage and 29 matched peptides, instead of 23% sequence coverage and 6 and 18 matched peptides as reported previously (Table 1). Similarly, the sequence coverage of apolipoprotein H (APOH_MOUSE) was increased by 17% with 5 additional peptides that could be mapped to this protein [7,9] (for sequence coverages, please refer to Additional file 3).

### Newly identified proteins

Thirteen new mouse serum proteins were identified with our approach. Among them, some were recently confirmed by another group [5].

In their approach [5], however, matched peptides were less on average when compared to our data. Apart from this difference, a total of 6 new proteins are reported by us (*italic font *in Table 2).

**Table 2:** New proteins, spectra interpretation and validation

**No**	**No Table 1**	**Swiss Prot entry**	**Swiss Prot accession**	**putative PTMs**	**Subcellular location**	**Mw (KDa)**	**pI**	**RMS error (ppm)**	**matrix**	**Swiss Prot**	**NCBInr**	**MSDB**	**Nr of matched peptides (Mascot)**	**Nr of matched peptides (Peptide Map)**
**1**	16	ANGL6 MOUSE	Q8R0Z6	G	Secreted; highly expressed in the liver	51.4	9	38	DHB	**56**	58	55	4	3
**2**	17	ANXA2 MOUSE	P07356	P	Secreted, extracellular space, extracellular matrix, basement membrane. Melanosome	38.8	8	45	CHCA	**82**	**81**	**82**	5	3
**3**	25	C1R MOUSE	Q8CG16	G	Secreted	81.5	5	15	DHB CHCA	**78**	**78**	**78**	8	5
**4**	32	CS1A MOUSE	Q8CG14	G	Predominantly expressed in liver	78.3	5	11	DHB	**66**	**66**	**66**	6	5
**5**	72	*LAC1 MOUSE*	P01843		Secreted	11.7	6	4	DHB	**70**	**70**	**70**	4	3
**6**	74	*ACTG MOUSE*	P63260	P	Cytoplasm, cytoskeleton	42.1	5	28	DHB CHCA	**188**	**188**	**188**	16	15
**7**	76	*CT160 MOUSE*	Q8VCC6			22	10	35	CHCA	**55**	56	56	5	4
**8**	77	CUL1 MOUSE	Q9WTX6	G	Embryo fibroblasts and embryo preadipocytes	90.3	8	49	CHCA	**62**	**62**	**62**	7	6
**9**	81	K2C5 MOUSE	Q922U2	P	Expressed in epidermis	62	8	34	DHB	**60**	60	60	11	11
**10**	85	*OST5 MOUSE*	Q8BSL4	G	Golgi apparatus membrane; Single-pass type II membrane protein	40.7	10	35	CHCA	**70**	**70**	**70**	6	5
**11**	87	*TPH1 MOUSE*	P17532	P	Cytoplasmatic enzyme, pineal gland	51.9	6	30	CHCA	**57**	57	57	7	5
**12**	88	KPYM MOUSE	P52480	P	Liver, red cells, muscles, brain	58	7	39	DHB	**53**	53	53	11	10
**13**	89	*DYN2 MOUSE*	P39054	P	Cytoplasm	98	7	51	DHB CHCA	**68**	**68**	**68**	9	9

We listed here the 13 proteins which re not present in the previous mouse serum maps [6-9,32,33].

The SwissProt entries *in Italic font *are proteins not identified in mouse serum proteome so far [5-9,32,33]. RMS (Root Mean Square) error is the calculated error for set of matched mass values (in ppm) in Mascot Search (matrix science, LTD, UK). It is measured in ppm. RMS error defines the limit of peptide mass tolerance (*Peptide tol +-*) for Mascot Peptide Mass Fingerprint to obtain a significant score (p < 0.05) of matched peptides to select protein entry.

The results were first searched in SwissProt databank and furthermore in NCBInr and MSDB databanks. The same criteria were applied (such as: mus musculus for the taxonomy, < +/- 50 ppm as peptide tolerance and one missed cleavage allowed for trypsin enzyme). **In bold **we highlighted the significant score (p < 0.05) according to the chosen database (SwissProt >53, NCBInr and MSDB >61) [21,51].

We also specified if one matrix or the combination of them allowed the identification.

Post translation modifications (PTMs). P: phosphorylation; G: Glycosylation.

For instance, we identified members of complement cascade activation, a positive mediator for angiogenesis (ANGL6_MOUSE), proteins specifically regulated in several tumor cells such as [KPYM_MOUSE] and proteins playing an important role in protein degradation and protein ubiquitinylation, whose altered activity could allow for abnormal cell proliferation (CUL-1_MOUSE). Notably, some of these newly identified proteins may serve as cancer biomarkers [5,41-43].

In this regard, we identified heparan sulfate glucosamine 3-O-sulfotransferase 5 (OST-5_MOUSE), a sulfotransferase that catalyzes the transfer of a sulfo group from the sulfo donor, 3'-phosphoadenosine-5'-phosphosulfate (PAPS), to the 3-OH position of a glucosamine unit of the HS. It displays anticoagulant activity by interacting with antithrombin (AT) protein through sulfanation at the 3-OH position of HS-saccharide [44-46]. From recent studies, new biological functions of activated HS have emerged, for instance in regulation of cancer growth and inflammatory responses [44,47].

Likewise, our identification of dynamin-2 (DYN2_MOUSE) in serum may be suggestive for extracellular matrix degradation, in case of invasive tumor cells (metastasis). Extensive studies are already published on the "invasive feet" enrichments in dynamin and actin and their involvement in extracellular matrix degradation in hepatocellular carcinoma [48-50].

## Conclusion

The combination of zoom in gels and the use of two different sample-matrix preparations in sequence improved protein identification of mouse serum proteins considerably and allowed for automated data acquisition as compared to previous methods using 2-DE and MALDI-MS (an example is given in Figure 4) [7,8]. Moreover, we obtained a higher number of matched peptides, as compared to sample pre-fractionation methods coupled with LC-MS/MS or other proteomic approaches (Table 1) [9,32,33]. Finally, we report an improved mouse serum proteome map and an identification of 13 proteins that were not reported in previous 2-DE studies. Even with the comprehensive study reported in [5], six of them are still novel (Table 2) [5-9,32,33]. We validated the newly identified proteins by repeating the searches against different databases (NCBInr and MSDB), as described above (Table 2) [51]. Moreover, the same spectra were further analysed with PeptideMap free software [22]. Altogether, identical results were obtained.

**Figure 5:**
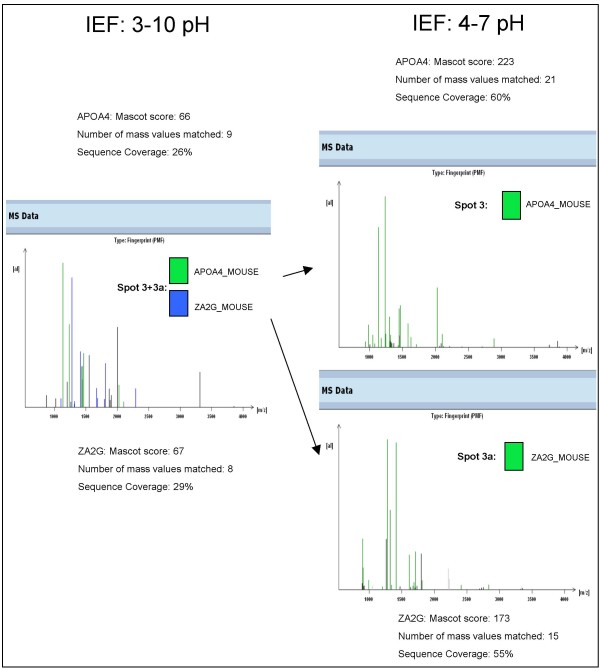
**Fast and reliable identification of mouse serum proteins**. We have depicted here an example of improvement of data acquisition by the use narrow-pH IPG strips for the IEF. The data of score and matched peptides were chosen from the best outcome in MALDI-MS analysis by both matrices (CHCA and DHB) (ProteinScape™ database).

There is a need for a reliable characterization of serum proteomes to enable biomarker discovery. In our opinion, the strength of this work lies in the right combination of protocols and its simplification. We report a simple and reliable identification of mouse serum protein (for instance, see Figure 5) and as shown in Table 1, our work evidences an improved identification when compared with previous studies based on pre-treatments of serum or other sophisticated methods.

Taken collectively, we view our protocol to be useful in biomarker discovery.

## List of abbreviations

ACN: acetonitril; DTT: dithiothreitol; IPG: immobilized pH gradient; MALDI-TOF-MS: Matrix Assisted Laser Desorption Ionization-Time of Flight-Mass Spectrometry; NH_4_HCO_3_: ammonium bicarbonate; SDS: Sodium Dodecyl Sulfate; TCA: trichloroacetic acid; TFA: trifluoracetic acid; TEMED: tetramethylenediamine.

## Competing interests

The authors declare that they have no competing interests.

## Authors' contributions

MSR designed and carried out the 2-DE and MALDI MS analysis. MSR and JB were responsible for the design of the study and were involved in writing the manuscript. All authors have read and approved the final manuscript.

## Supplementary Material

Additional file 1 **Validation of the new protein entries**. We analysed the same spectra with Mascot search engine in ProteinScape™) and PeptideMap (PROWL free softwares). The ionized tryptic peptides resulted matched to the same protein sequences. Click here for file

Additional file 2 **Mascot and PeptideMap**. Single entries from both Mascot and PeptideMap for the new identifications. Click here for file

Additional file 3 **Protein sequences**. We listed all identified protein sequence, the PMF (MS analysis) and PFF (tandem-MS analysis). We chose representative tandem MS for every ion peak fitting with the protein sequence. Click here for file
