# Supplementary material for: A simple and reliable protocol for mouse serum proteome profiling studies by use of two-dimensional electrophoresis and MALDI TOF/TOF mass spectrometry
Source: Proteome Sci. 2008 Sep 12;6:25. doi: 10.1186/1477-5956-6-25 (PMC2563006; doi:10.1186/1477-5956-6-25)
Supplement: Additional file 1 — Validation of the new protein entries. We analysed the same spectra with Mascot search engine in ProteinScape™) and PeptideMap (PROWL free softwares). The ionized tryptic peptides resulted matched to the same protein sequences. [file 1477-5956-6-25-S1.ppt]

## Slide 1
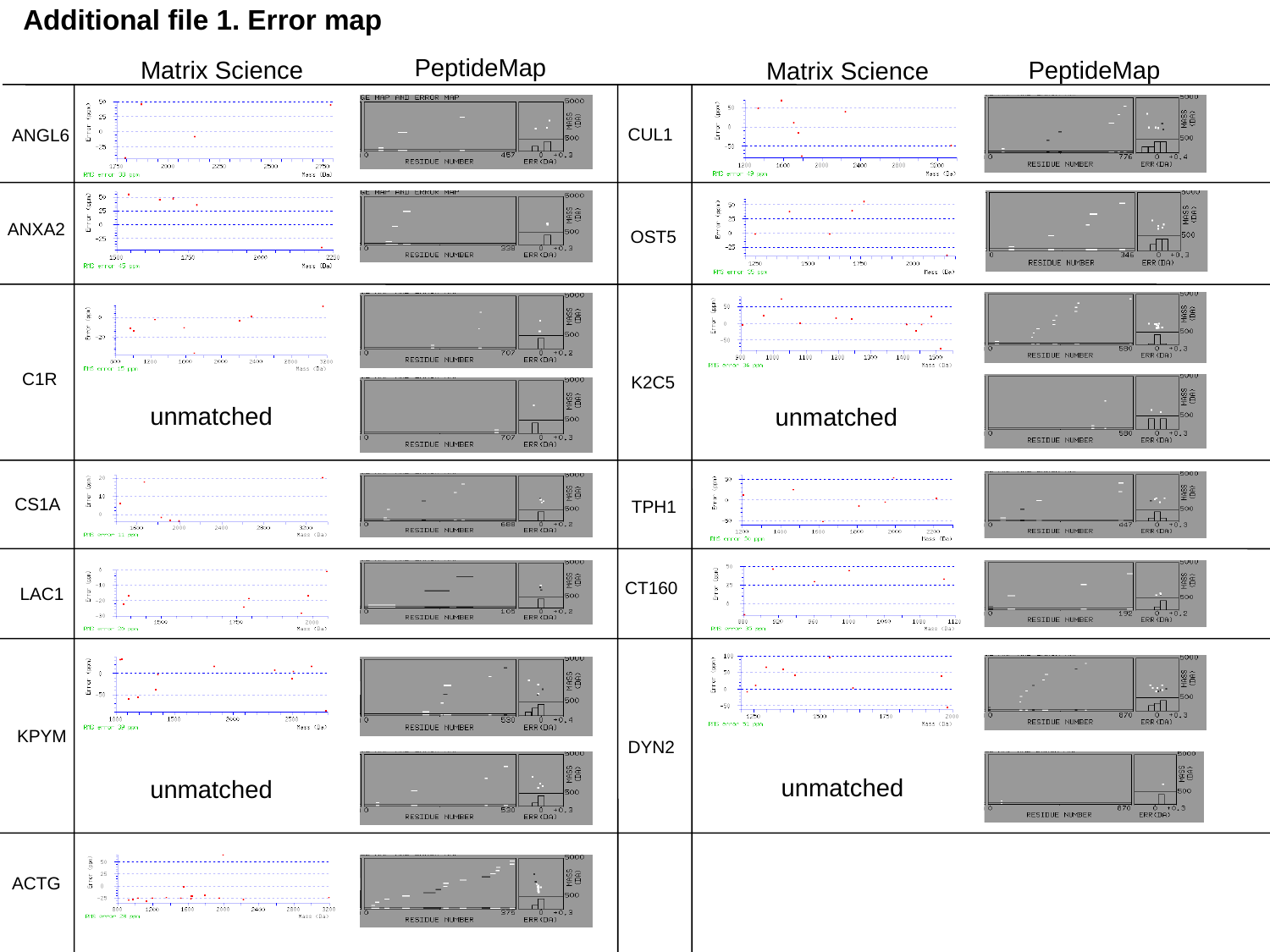

Additional file 1. Error map
PeptideMap
Matrix Science
PeptideMap
Matrix Science
CUL1
ANGL6
ANXA2
OST5
C1R
K2C5
unmatched
unmatched
CS1A
TPH1
CT160
LAC1
KPYM
DYN2
unmatched
unmatched
ACTG
