# Supplementary material for: A simple and reliable protocol for mouse serum proteome profiling studies by use of two-dimensional electrophoresis and MALDI TOF/TOF mass spectrometry
Source: Proteome Sci. 2008 Sep 12;6:25. doi: 10.1186/1477-5956-6-25 (PMC2563006; doi:10.1186/1477-5956-6-25)
Supplement: Additional file 2 — Mascot and PeptideMap. Single entries from both Mascot and PeptideMap for the new identifications. [file 1477-5956-6-25-S2.doc]

| | |  | **PeptideMap - Results** | |  | | --- | |  | |  | | | --- | --- | --- | --- | --- | --- | | | --- | --- | --- | --- | --- | --- | --- | |
| --- | --- | --- | --- | --- | --- | --- | --- |
| | | **[Name: (Q8R0Z6) Angiopoietin-related protein 6](http://prowl.rockefeller.edu/prowl/pmhelp.htm#PROTEIN_NAME)** | | --- | | | --- | --- | | Number of Measured Peptides : 4  Number of Matched Peptides  : 3  Min. Coverage of Sequence   : 13% | | |  | | --- |  | Measured | Ave/ | Computed | Error | Residues | Missed |  | | --- | --- | --- | --- | --- | --- | --- | | Mass | Mono | Mass | (Da) | | Start | End | | --- | --- | | Cut | Peptide Sequence | | 1793.778 | M | 1793.857 | -0.079 | | 45 | 60 | | --- | --- | | 1 | SSEATQDSELATLRMR | | 1869.928 | M | 1869.843 | 0.085 | | 290 | 304 | | --- | --- | | 0 | QDGSVNFFTNWQHYK | | 2787.574 | M | 2787.451 | 0.123 | | 104 | 130 | | --- | --- | | 1 | AQLQQEARAEPDLGAEPAAA LGLLAER | | | | **Unmatched Monoisotopic Masses:** 2127.900 | | --- | | | | **Input Summary** | | --- | | | Protein | 1  *    10   *    20   *    30   *    40   *    50      1 MGTARLRKLQLLLLLGAWRALGGAARCRVTLVLSPQKATSAVCRSSEATQ    51 DSELATLRMRLGRHEELLRALQRRAAEGGALADEVRALREHSLTLNTRLG   101 QLRAQLQQEARAEPDLGAEPAAALGLLAERALDAEAEARRTTARLQQLDA   151 QLREHAQLMSQHSSLLGRLQRACAGPERGQQQVLPLPLAPLVPLSLVGSA   201 SNTSRRLDQTPEHQREQSLRQQGPPSSLLPTGHLAVPTRPVGPWRDCAEA   251 HGAGHWQSGVYDLRLGRRVVAVWCEQQQEGGGWTVIQRRQDGSVNFFTNW   301 QHYKAGFGRPEGEYWLGLEPVHQVTSRGDHELLILLEDWGGRAARAHYDS   351 FSLEPESDHYRLRLGQYHGDAGDSLSWHNDKPFSTVDRDRDSYSGNCALY   401 HRGGWWYHACAHSNLNGVWYHGGHYRSRYQDGVYWAEFRGGAYSLKKAVM   451 LTRLVRL | | --- | --- | | | | Enzyme | | Name | Site(s) | Terminal | Mod. | Proline | Missed Cut | | --- | --- | --- | --- | --- | --- | | Trypsin | KR | C |  | N | 2 | | | --- | --- | --- | --- | --- | --- | --- | --- | --- | --- | --- | --- | --- | --- | | | | Global Modification | | Name | Site(s) | Formula | Min. Occ. | Max. Occ. | | --- | --- | --- | --- | --- | | Oxidation (M) | M | +O | Partial | | | | --- | --- | --- | --- | --- | --- | --- | --- | --- | --- | --- | --- | | | | Monoisotopic Masses  (+/- 50 ppm) | 1793.778 2787.574 2127.900 1869.928 | | --- | --- | | | | Charge State | MH+ | | --- | --- | | | |
| | | **[Name: (P07356) Annexin A2](http://prowl.rockefeller.edu/prowl/pmhelp.htm#PROTEIN_NAME)** | | --- | | | --- | --- | | Number of Measured Peptides : 5  Number of Matched Peptides  : 3  Min. Coverage of Sequence   : 13% | | |  | | --- |  | Measured | Ave/ | Computed | Error | Residues | Missed |  | | --- | --- | --- | --- | --- | --- | --- | | Mass | Mono | Mass | (Da) | | Start | End | | --- | --- | | Cut | Peptide Sequence | | 1541.926 | M | 1541.841 | 0.085 | | 49 | 62 | | --- | --- | | 0 | GVDEVTIVNILTNR | | 1650.045 | M | 1649.971 | 0.074 | | 88 | 103 | | --- | --- | | 0 | SALSGHLETVILGLLK | | 1694.929 | M | 1694.848 | 0.081 | | 63 | 76 | | --- | --- | | 1 | SNVQRQDIAFAYQR | | | | **Unmatched Monoisotopic Masses:** 2209.965 1776.919 | | --- | | | | **Input Summary** | | --- | | | Protein | 1  *    10   *    20   *    30   *    40   *    50      1 STVHEILCKLSLEGDHSTPPSAYGSVKPYTNFDAERDALNIETAVKTKGV    51 DEVTIVNILTNRSNVQRQDIAFAYQRRTKKELPSALKSALSGHLETVILG   101 LLKTPAQYDASELKASMKGLGTDEDSLIEIICSRTNQELQEINRVYKEMY   151 KTDLEKDIISDTSGDFRKLMVALAKGRRAEDGSVIDYELIDQDARELYDA   201 GVKRKGTDVPKWISIMTERSVCHLQKVFERYKSYSPYDMLESIKKEVKGD   251 LENAFLNLVQCIQNKPLYFADRLYDSMKGKGTRDKVLIRIMVSRSEVDML   301 KIRSEFKRKYGKSLYYYIQQDTKGDYQKALLYLCGGDD | | --- | --- | | | | Enzyme | | Name | Site(s) | Terminal | Mod. | Proline | Missed Cut | | --- | --- | --- | --- | --- | --- | | Trypsin | KR | C |  | N | 2 | | | --- | --- | --- | --- | --- | --- | --- | --- | --- | --- | --- | --- | --- | --- | | | | Global Modification | | Name | Site(s) | Formula | Min. Occ. | Max. Occ. | | --- | --- | --- | --- | --- | | Oxidation (M) | M | +O | Partial | | | | --- | --- | --- | --- | --- | --- | --- | --- | --- | --- | --- | --- | | | | Monoisotopic Masses  (+/- 100 ppm) | 1541.926 1694.929 1650.045 2209.965 1776.919 | | --- | --- | | | | Charge State | MH+ | | --- | --- | | | |
| | |  |  |  | | --- | --- | --- | | | --- | --- | --- | --- | |
| | | **[Name: (Q8CG16) Complement C1r-A subcomponent precursor](http://prowl.rockefeller.edu/prowl/pmhelp.htm#PROTEIN_NAME)** | | --- | | | --- | --- | | Number of Measured Peptides : 8  Number of Matched Peptides  : 3  Min. Coverage of Sequence   : 5% | | |  | | --- |  | Measured | Ave/ | Computed | Error | Residues | Missed |  | | --- | --- | --- | --- | --- | --- | --- | | Mass | Mono | Mass | (Da) | | Start | End | | --- | --- | | Cut | Peptide Sequence | | 970.546 | M | 970.557 | -0.011 | | 534 | 541 | | --- | --- | | 1 | LGHHPVRR | | 1011.535 | M | 1011.550 | -0.015 | | 542 | 549 | | --- | --- | | 0 | VIIHPDYR | | 2208.077 | M | 2208.085 | -0.008 | | 313 | 330 | | --- | --- | | 0 | ALDEFTIIQDPQPQYQFR | | | | **Unmatched Monoisotopic Masses:** 3155.485 1578.667 2345.043 1695.817 1244.578 | | --- | | | | **Input Summary** | | --- | | | Protein | 1  *    10   *    20   *    30   *    40   *    50      1 MWLFALLVTLFYGVEGSIYLPQKLYGEVTSPLYPKPYPSDLETTTVITVP    51 MGYRVKLVFWQFDVEPSEGCFYDYVKISADKQTLGRFCGQLDSPLGNPPG   101 SKEFMSQGNKMLLTFHTDFSNEENGTIMFYKGFLAYYQAVDLDECASQPN   151 SVEEGLQPRCQHLCHNYVGGYFCSCHPGYELQKDGQSCQAECSSELYTEP   201 SGYVSSLEYPQPYPPDLRCNYSIRVERGLTVHLKFLDPFEIDDHQQVHCP   251 YDQLQIYANGKNLGEFCGKQRPPDLDTSSNAVDLLFFTDESGDSRGWKLH   301 YTTETIKCPQPKALDEFTIIQDPQPQYQFRDYFIVTCKQGYQLMEGNQAL   351 LSFTAVCQNDGTWHRAMPRCKIKNCGQPQSLSNGDFRYITTKGVTTYEAS   401 IQYHCHEPYYKMLTRAGSSESMRGIYTCTAQGIWKNEEEGEKMPRCLPVC   451 GKPVNPVTQKERIIRGQPARPGNFPWQAFTTTHGRGGGALLGDRWILTAA   501 HTIYPKHHNKENDNANPKMLVFLGHTNVEQIKKLGHHPVRRVIIHPDYRQ   551 DEPNNFEGDIALLELENSVTLGPELLPICLPDNETFYGQGLMGYVSGFGI   601 TEDKLAFDLRFVRLPVADSEACQRWLQTKKDTSPFSQNMFCSGDPAVQQD   651 ACQGDSGGVFAVRDRNRDIWVATGIVSWGIGCGEGYGFYTKVLNYVDWIK   701 KEMGDEN | | --- | --- | | | | Enzyme | | Name | Site(s) | Terminal | Mod. | Proline | Missed Cut | | --- | --- | --- | --- | --- | --- | | Trypsin | KR | C |  | N | 2 | | | --- | --- | --- | --- | --- | --- | --- | --- | --- | --- | --- | --- | --- | --- | | | | Global Modification | | Name | Site(s) | Formula | Min. Occ. | Max. Occ. | | --- | --- | --- | --- | --- | | Oxidation (M) | M | +O | Partial | | | | --- | --- | --- | --- | --- | --- | --- | --- | --- | --- | --- | --- | | | | Monoisotopic Masses  (+/- 100 ppm) | 3155.485 2208.077 1578.667 2345.043 1695.817 970.546 1011.535 1244.578 | | --- | --- | | | | Charge State | MH+ | | --- | --- | | | |
| | | **[Name: (Q8CG16) Complement C1r-A subcomponent precursor](http://prowl.rockefeller.edu/prowl/pmhelp.htm#PROTEIN_NAME)** | | --- | | | --- | --- | | Number of Measured Peptides : 11  Number of Matched Peptides  : 1  Min. Coverage of Sequence   : 3% | | |  | | --- |  | Measured | Ave/ | Computed | Error | Residues | Missed |  | | --- | --- | --- | --- | --- | --- | --- | | Mass | Mono | Mass | (Da) | | Start | End | | --- | --- | | Cut | Peptide Sequence | | 2246.067 | M | 2246.163 | -0.096 | | 611 | 629 | | --- | --- | | 2 | FVRLPVADSEACQRWLQTK | | | | **Unmatched Monoisotopic Masses:** 1188.567 1319.581 1329.745 1478.757 1496.740 1598.762 1908.930 2226.073 2230.071 2232.068 | | --- | | | | **Input Summary** | | --- | | | Protein | 1  *    10   *    20   *    30   *    40   *    50      1 MWLFALLVTLFYGVEGSIYLPQKLYGEVTSPLYPKPYPSDLETTTVITVP    51 MGYRVKLVFWQFDVEPSEGCFYDYVKISADKQTLGRFCGQLDSPLGNPPG   101 SKEFMSQGNKMLLTFHTDFSNEENGTIMFYKGFLAYYQAVDLDECASQPN   151 SVEEGLQPRCQHLCHNYVGGYFCSCHPGYELQKDGQSCQAECSSELYTEP   201 SGYVSSLEYPQPYPPDLRCNYSIRVERGLTVHLKFLDPFEIDDHQQVHCP   251 YDQLQIYANGKNLGEFCGKQRPPDLDTSSNAVDLLFFTDESGDSRGWKLH   301 YTTETIKCPQPKALDEFTIIQDPQPQYQFRDYFIVTCKQGYQLMEGNQAL   351 LSFTAVCQNDGTWHRAMPRCKIKNCGQPQSLSNGDFRYITTKGVTTYEAS   401 IQYHCHEPYYKMLTRAGSSESMRGIYTCTAQGIWKNEEEGEKMPRCLPVC   451 GKPVNPVTQKERIIRGQPARPGNFPWQAFTTTHGRGGGALLGDRWILTAA   501 HTIYPKHHNKENDNANPKMLVFLGHTNVEQIKKLGHHPVRRVIIHPDYRQ   551 DEPNNFEGDIALLELENSVTLGPELLPICLPDNETFYGQGLMGYVSGFGI   601 TEDKLAFDLRFVRLPVADSEACQRWLQTKKDTSPFSQNMFCSGDPAVQQD   651 ACQGDSGGVFAVRDRNRDIWVATGIVSWGIGCGEGYGFYTKVLNYVDWIK   701 KEMGDEN | | --- | --- | | | | Enzyme | | Name | Site(s) | Terminal | Mod. | Proline | Missed Cut | | --- | --- | --- | --- | --- | --- | | Trypsin | KR | C |  | N | 2 | | | --- | --- | --- | --- | --- | --- | --- | --- | --- | --- | --- | --- | --- | --- | | | | Global Modification | | Name | Site(s) | Formula | Min. Occ. | Max. Occ. | | --- | --- | --- | --- | --- | | Oxidation (M) | M | +O | Partial | | | | --- | --- | --- | --- | --- | --- | --- | --- | --- | --- | --- | --- | | | | Monoisotopic Masses  (+/- 100 ppm) | 1188.567 1319.581 1329.745 1478.757 1496.740 1598.762 1908.930 2226.073 2230.071 2232.068 2246.067 | | --- | --- | | | | Charge State | MH+ | | --- | --- | | | |
| | | **[Name: (Q8CG14) Complement C1s-A subcomponent precursor](http://prowl.rockefeller.edu/prowl/pmhelp.htm#PROTEIN_NAME)** | | --- | | | --- | --- | | Number of Measured Peptides : 6  Number of Matched Peptides  : 5  Min. Coverage of Sequence   : 11% | | |  | | --- |  | Measured | Ave/ | Computed | Error | Residues | Missed |  | | --- | --- | --- | --- | --- | --- | --- | | Mass | Mono | Mass | (Da) | | Start | End | | --- | --- | | Cut | Peptide Sequence | | 1438.765 | M | 1438.756 | 0.009 | | 409 | 420 | | --- | --- | | 0 | WVNDQLGIELPR | | 1669.823 | M | 1669.794 | 0.029 | | 106 | 119 | | --- | --- | | 0 | LQVVFTSDFSNEER | | 1829.896 | M | 1829.900 | -0.004 | | 446 | 459 | | --- | --- | | 0 | IENFPWQVFFNHPR | | 1908.918 | M | 1908.925 | -0.007 | | 90 | 105 | | --- | --- | | 0 | SPNSPIIEEFQFPYNK | | 1995.985 | M | 1995.993 | -0.008 | | 263 | 280 | | --- | --- | | 0 | TQSNTLGIVFQTDLMGQK  (1)+O@M | | | | **Unmatched Monoisotopic Masses:** 3352.662 | | --- | | | | **Input Summary** | | --- | | | Protein | 1  *    10   *    20   *    30   *    40   *    50      1 MWCLVLFSLLASFSAEPTMHGEILSPNYPQAYPNDVVKSWDIEVPEGFGI    51 HLYFTHVDIEPSESCAYDSVQIISGGIEEGRLCGQKTSKSPNSPIIEEFQ   101 FPYNKLQVVFTSDFSNEERFTGFAAYYTAIDINECTDFTDVPCSHFCNNF   151 IGGYFCSCPPEYFLHDDMRNCGVNCSGDVFTALIGEISSPNYPNPYPENS   201 RCEYQIQLQEGFQVVVTMQREDFDVEPADSEGNCPDSLTFASKNQQFGPY   251 CGNGFPGPLTIRTQSNTLGIVFQTDLMGQKKGWKLRYHGDPISCAKKITA   301 NSTWEPDKAKYVFKDVVKITCVDGFEVVEGHVSSTSYYSTCQSDGQWSNS   351 GLKCQPVYCGIPDPIANGKVEEPENSVFGTVVHYTCEEPYYYMEHEEGGE   401 YRCAANGRWVNDQLGIELPRCIPACGVPTEPFQVHQRIFGGQPAKIENFP   451 WQVFFNHPRASGALINEYWVLTAAHVLEKISDPLMYVGTMSVRTTLLENA   501 QRLYSKRVFIHPSWKKEDDPNTRTNFDNDIALVQLKDPVKMGPKVSPICL   551 PGTSSEYNVSPGDMGLISGWGSTEKKVFVINLRGAKVPVTSLETCKQVKE   601 ENPTVRPEDYVFTDNMICAGEKGVDSCHGDSGGAFAFQVPNVTVPKFYVA   651 GLVSWGKRCGTYGVYTKVKNYVDWILKTMQENSGPRKD | | --- | --- | | | | Enzyme | | Name | Site(s) | Terminal | Mod. | Proline | Missed Cut | | --- | --- | --- | --- | --- | --- | | Trypsin | KR | C |  | N | 2 | | | --- | --- | --- | --- | --- | --- | --- | --- | --- | --- | --- | --- | --- | --- | | | | Global Modification | | Name | Site(s) | Formula | Min. Occ. | Max. Occ. | | --- | --- | --- | --- | --- | | Oxidation (M) | M | +O | Partial | | | | --- | --- | --- | --- | --- | --- | --- | --- | --- | --- | --- | --- | | | | Monoisotopic Masses  (+/- 50 ppm) | 1908.918 1669.823 1995.985 1438.765 1829.896 3352.662 | | --- | --- | | | | Charge State | MH+ | | --- | --- | | | |
| | | **[Name: (P01843) Ig lambda-1 chain C region](http://prowl.rockefeller.edu/prowl/pmhelp.htm#PROTEIN_NAME)** | | --- | | | --- | --- | | Number of Measured Peptides : 4  Number of Matched Peptides  : 3  Min. Coverage of Sequence   : 46% | | |  | | --- |  | Measured | Ave/ | Computed | Error | Residues | Missed |  | | --- | --- | --- | --- | --- | --- | --- | | Mass | Mono | Mass | (Da) | | Start | End | | --- | --- | | Cut | Peptide Sequence | | 1391.679 | M | 1391.675 | 0.004 | | 65 | 76 | | --- | --- | | 0 | YMASSYLTLTAR  (1)+O@M | | 1790.826 | M | 1790.835 | -0.009 | | 43 | 59 | | --- | --- | | 0 | VDGTPVTQGMETTQPSK  (1)+O@M | | 2047.989 | M | 2047.994 | -0.005 | | 4 | 22 | | --- | --- | | 0 | SSPSVTLFPPSSEELETNK | | | | **Unmatched Monoisotopic Masses:** 1984.882 | | --- | | | | **Input Summary** | | --- | | | Protein | 1  *    10   *    20   *    30   *    40   *    50      1 QPKSSPSVTLFPPSSEELETNKATLVCTITDFYPGVVTVDWKVDGTPVTQ    51 GMETTQPSKQSNNKYMASSYLTLTARAWERHSSYSCQVTHEGHTVEKSLS   101 RADCS | | --- | --- | | | | Enzyme | | Name | Site(s) | Terminal | Mod. | Proline | Missed Cut | | --- | --- | --- | --- | --- | --- | | Trypsin | KR | C |  | N | 2 | | | --- | --- | --- | --- | --- | --- | --- | --- | --- | --- | --- | --- | --- | --- | | | | Global Modification | | Name | Site(s) | Formula | Min. Occ. | Max. Occ. | | --- | --- | --- | --- | --- | | Oxidation (M) | M | +O | Partial | | | | --- | --- | --- | --- | --- | --- | --- | --- | --- | --- | --- | --- | | | | Monoisotopic Masses  (+/- 50 ppm) | 2047.989 1790.826 1391.679 1984.882 | | --- | --- | | | | Charge State | MH+ | | --- | --- | | | |
| | | **[Name: (P63260) Actin, cytoplasmic 2](http://prowl.rockefeller.edu/prowl/pmhelp.htm#PROTEIN_NAME)** | | --- | | | --- | --- | | Number of Measured Peptides : 16  Number of Matched Peptides  : 15  Min. Coverage of Sequence   : 49% | | |  | | --- |  | Measured | Ave/ | Computed | Error | Residues | Missed |  | | --- | --- | --- | --- | --- | --- | --- | | Mass | Mono | Mass | (Da) | | Start | End | | --- | --- | | Cut | Peptide Sequence | | 922.531 | M | 922.560 | -0.029 | | 329 | 336 | | --- | --- | | 1 | IIAPPERK | | 975.412 | M | 975.441 | -0.029 | | 19 | 28 | | --- | --- | | 0 | AGFAGDDAPR | | 1035.617 | M | 1035.644 | -0.027 | | 327 | 335 | | --- | --- | | 1 | IKIIAPPER | | 1131.483 | M | 1131.519 | -0.036 | | 197 | 206 | | --- | --- | | 0 | GYSFTTTAER | | 1197.667 | M | 1197.698 | -0.031 | | 29 | 39 | | --- | --- | | 0 | AVFPSIVGRPR | | 1353.583 | M | 1353.616 | -0.033 | | 51 | 62 | | --- | --- | | 1 | DSYVGDEAQSKR | | 1515.657 | M | 1515.695 | -0.038 | | 360 | 372 | | --- | --- | | 0 | QEYDESGPSIVHR | | 1547.802 | M | 1547.805 | -0.003 | | 313 | 326 | | --- | --- | | 1 | MQKEITALAPSTMK | | 1628.773 | M | 1628.815 | -0.042 | | 197 | 210 | | --- | --- | | 1 | GYSFTTTAEREIVR | | 1638.794 | M | 1638.828 | -0.034 | | 178 | 191 | | --- | --- | | 1 | LDLAGRDLTDYLMK  (1)+O@M | | 1643.755 | M | 1643.790 | -0.035 | | 360 | 373 | | --- | --- | | 1 | QEYDESGPSIVHRK | | 1789.850 | M | 1789.884 | -0.034 | | 239 | 254 | | --- | --- | | 0 | SYELPDGQVITIGNER | | 1953.008 | M | 1953.057 | -0.049 | | 96 | 113 | | --- | --- | | 0 | VAPEEHPVLLTEAPLNPK | | 2229.994 | M | 2230.057 | -0.063 | | 292 | 312 | | --- | --- | | 0 | DLYANTVLSGGTTMYPGIAD R  (1)+O@M | | 3198.523 | M | 3198.601 | -0.078 | | 148 | 177 | | --- | --- | | 0 | TTGIVMDSGDGVTHTVPIYE GYALPHAILR  (1)+O@M | | | | **Unmatched Monoisotopic Masses:** 1998.003 | | --- | | | | **Input Summary** | | --- | | | Protein | 1  *    10   *    20   *    30   *    40   *    50      1 MEEEIAALVIDNGSGMCKAGFAGDDAPRAVFPSIVGRPRHQGVMVGMGQK    51 DSYVGDEAQSKRGILTLKYPIEHGIVTNWDDMEKIWHHTFYNELRVAPEE   101 HPVLLTEAPLNPKANREKMTQIMFETFNTPAMYVAIQAVLSLYASGRTTG   151 IVMDSGDGVTHTVPIYEGYALPHAILRLDLAGRDLTDYLMKILTERGYSF   201 TTTAEREIVRDIKEKLCYVALDFEQEMATAASSSSLEKSYELPDGQVITI   251 GNERFRCPEALFQPSFLGMESCGIHETTFNSIMKCDVDIRKDLYANTVLS   301 GGTTMYPGIADRMQKEITALAPSTMKIKIIAPPERKYSVWIGGSILASLS   351 TFQQMWISKQEYDESGPSIVHRKCF | | --- | --- | | | | Enzyme | | Name | Site(s) | Terminal | Mod. | Proline | Missed Cut | | --- | --- | --- | --- | --- | --- | | Trypsin | KR | C |  | N | 2 | | | --- | --- | --- | --- | --- | --- | --- | --- | --- | --- | --- | --- | --- | --- | | | | Global Modification | | Name | Site(s) | Formula | Min. Occ. | Max. Occ. | | --- | --- | --- | --- | --- | | Oxidation (M) | M | +O | Partial | | | | --- | --- | --- | --- | --- | --- | --- | --- | --- | --- | --- | --- | | | | Monoisotopic Masses  (+/- 50 ppm) | 1998.003 975.412 1197.667 1353.583 1953.008 3198.523 1638.794 1131.483 1628.773 1789.850 2229.994 1547.802 1035.617 922.531 1515.657 1643.755 | | --- | --- | | | | Charge State | MH+ | | --- | --- | | | |
| | | **[Name: (Q9WTX6) Cullin-1 (CUL-1)](http://prowl.rockefeller.edu/prowl/pmhelp.htm#PROTEIN_NAME)** | | --- | | | --- | --- | | Number of Measured Peptides : 7  Number of Matched Peptides  : 6  Min. Coverage of Sequence   : 14% | | |  | | --- |  | Measured | Ave/ | Computed | Error | Residues | Missed |  | | --- | --- | --- | --- | --- | --- | --- | | Mass | Mono | Mass | (Da) | | Start | End | | --- | --- | | Cut | Peptide Sequence | | 1581.865 | M | 1581.756 | 0.109 | | 316 | 328 | | --- | --- | | 1 | NEDLGRMYNLVSR  (1)+O@M | | 1707.945 | M | 1707.926 | 0.019 | | 727 | 741 | | --- | --- | | 0 | HQQLLGEVLTQLSSR | | 1755.774 | M | 1755.802 | -0.028 | | 694 | 707 | | --- | --- | | 1 | QEQETTHKNIEEDR | | 1791.815 | M | 1791.951 | -0.136 | | 74 | 90 | | --- | --- | | 1 | KGQTPGGAQFVGLELYK | | 2246.155 | M | 2246.067 | 0.088 | | 377 | 397 | | --- | --- | | 0 | YNALVMSAFNNDAGFVAALD K  (1)+O@M | | 3345.631 | M | 3345.795 | -0.164 | | 648 | 676 | | --- | --- | | 1 | LLVLEDENANVDEVELKPDT LIKLYLGYK | | | | **Unmatched Monoisotopic Masses:** 1336.720 | | --- | | | | **Input Summary** | | --- | | | Protein | 1  *    10   *    20   *    30   *    40   *    50      1 MSSNRSQNPHGLKQIGLDQIWDDLRAGIQQVYTRQSMAKSRYMELYTHVY    51 NYCTSVHQSNQARGAGVPPSKSKKGQTPGGAQFVGLELYKRLKEFLKNYL   101 TNLLKDGEDLMDESVLKFYTQQWEDYRFSSKVLNGICAYLNRHWVRRECD   151 EGRKGIYEIYSLALVTWRDCLFRPLNKQVTNAVLKLIEKERNGETINTRL   201 ISGVVQSYVELGLNEDDAFAKGPTLTVYKESFESQFLADTERFYTRESTE   251 FLQQNPVTEYMKKAEARLLEEQRRVQVYLHESTQDELARKCEQVLIEKHL   301 EIFHTEFQNLLDADKNEDLGRMYNLVSRIQDGLGELKKLLETHIHNQGLA   351 AIEKCGEAALNDPKMYVQTVLDVHKKYNALVMSAFNNDAGFVAALDKACG   401 RFINNNAVTKMAQSSSKSPELLARYCDSLLKKSSKNPEEAELEDTLNQVM   451 VVFKYIEDKDVFQKFYAKMLAKRLVHQNSASDDAEASMISKLKQACGFEY   501 TSKLQRMFQDIGVSKDLNEQFKKHLTNSEPLDLDFSIQVLSSGSWPFQQS   551 CTFALPSELERSYQRFTAFYASRHSGRKLTWLYQLSKGELVTNCFKNRYT   601 LQASTFQMAILLQYNTEDAYTVQQLTDSTQIKMDILAQVLQILLKSKLLV   651 LEDENANVDEVELKPDTLIKLYLGYKNKKLRVNINVPMKTEQKQEQETTH   701 KNIEEDRKLLIQAAIVRIMKMRKVLKHQQLLGEVLTQLSSRFKPRVPVIK   751 KCIDILIEKEYLERVDGEKDTYSYLA | | --- | --- | | | | Enzyme | | Name | Site(s) | Terminal | Mod. | Proline | Missed Cut | | --- | --- | --- | --- | --- | --- | | Trypsin | KR | C |  | N | 2 | | | --- | --- | --- | --- | --- | --- | --- | --- | --- | --- | --- | --- | --- | --- | | | | Global Modification | | Name | Site(s) | Formula | Min. Occ. | Max. Occ. | | --- | --- | --- | --- | --- | | Oxidation (M) | M | +O | Partial | | | | --- | --- | --- | --- | --- | --- | --- | --- | --- | --- | --- | --- | | | | Monoisotopic Masses  (+/- 100 ppm) | 1791.815 1581.865 2246.155 1336.720 3345.631 1755.774 1707.945 | | --- | --- | | | | Charge State | MH+ | | --- | --- | | | |
| | | **[Name: (Q8BSL4) Heparan sulfate glucosamine 3-O-sulfotransferase 5](http://prowl.rockefeller.edu/prowl/pmhelp.htm#PROTEIN_NAME)** | | --- | | | --- | --- | | Number of Measured Peptides : 6  Number of Matched Peptides  : 5  Min. Coverage of Sequence   : 20% | | |  | | --- |  | Measured | Ave/ | Computed | Error | Residues | Missed |  | | --- | --- | --- | --- | --- | --- | --- | | Mass | Mono | Mass | (Da) | | Start | End | | --- | --- | | Cut | Peptide Sequence | | 1246.665 | M | 1246.666 | -0.001 | | 231 | 240 | | --- | --- | | 1 | TSIYTKHLER | | 1408.773 | M | 1408.721 | 0.052 | | 47 | 59 | | --- | --- | | 0 | FGGAHNQAELPLR | | 1602.907 | M | 1602.909 | -0.002 | | 312 | 325 | | --- | --- | | 1 | IHPEVDPSVITKLR | | 1708.953 | M | 1708.885 | 0.068 | | 142 | 155 | | --- | --- | | 1 | KMPFSYPQQITIEK | | 2162.011 | M | 2162.094 | -0.083 | | 241 | 257 | | --- | --- | | 1 | WLKYFPIEQFHIVDGDR | | | | **Unmatched Monoisotopic Masses:** 1764.968 | | --- | | | | **Input Summary** | | --- | | | Protein | 1  *    10   *    20   *    30   *    40   *    50      1 MLFKQQVWLRQKLLVLGSLAVGSLLYLVARVGSLDRLQPICPVESRFGGA    51 HNQAELPLRALQFKRGLLHEFRKGNSSKEQVHLHDLVQQLPKAIIIGVRK   101 GGTRALLEMLNLHPAVVKASQEIHFFDNDENYAKGIEWYRKKMPFSYPQQ   151 ITIEKSPAYFITEEVPERIYKMNSSIKLLIIVREPTTRAISDYTQVLEGK   201 ERKNKTYYKFEKLAIDPNTCEVNTKYKAVRTSIYTKHLERWLKYFPIEQF   251 HIVDGDRLITEPLPELQLVEKFLNLPPRISQYNLYFNATRGFYCLRFNII   301 FNKCLAGSKGRIHPEVDPSVITKLRKFFHPFNQKFYQITGRTLNWP | | --- | --- | | | | Enzyme | | Name | Site(s) | Terminal | Mod. | Proline | Missed Cut | | --- | --- | --- | --- | --- | --- | | Trypsin | KR | C |  | N | 2 | | | --- | --- | --- | --- | --- | --- | --- | --- | --- | --- | --- | --- | --- | --- | | | | Global Modification | | Name | Site(s) | Formula | Min. Occ. | Max. Occ. | | --- | --- | --- | --- | --- | | Oxidation (M) | M | +O | Partial | | | | --- | --- | --- | --- | --- | --- | --- | --- | --- | --- | --- | --- | | | | Monoisotopic Masses  (+/- 50 ppm) | 1408.773 1708.953 1764.968 1246.665 2162.011 1602.907 | | --- | --- | | | | Charge State | MH+ | | --- | --- | | | |
| | | [Name: (Q922U2) Keratin, type II cytoskeletal 5](http://prowl.rockefeller.edu/prowl/pmhelp.htm#PROTEIN_NAME) | | --- | | | --- | --- | | Number of Measured Peptides : 11  Number of Matched Peptides  : 11  Min. Coverage of Sequence   : 17% | | |  | | --- |  | Measured | Ave/ | Computed | Error | Residues | Missed |  | | --- | --- | --- | --- | --- | --- | --- | | Mass | Mono | Mass | (Da) | | Start | End | | --- | --- | | Cut | Peptide Sequence | | 905.456 | M | 905.460 | -0.004 | | 182 | 188 | | --- | --- | | 0 | FLEQQNK | | 970.493 | M | 970.471 | 0.022 | | 567 | 575 | | --- | --- | | 0 | FVSTTSSSR | | 1025.522 | M | 1025.448 | 0.074 | | 272 | 280 | | --- | --- | | 0 | DVDAAYMNK | | 1081.590 | M | 1081.592 | -0.002 | | 173 | 181 | | --- | --- | | 1 | FASFIDKVR | | 1193.584 | M | 1193.567 | 0.017 | | 359 | 368 | | --- | --- | | 0 | YEELQQTAGR | | 1241.572 | M | 1241.556 | 0.016 | | 349 | 358 | | --- | --- | | 0 | TEAESWYQTK | | 1409.717 | M | 1409.722 | -0.005 | | 260 | 271 | | --- | --- | | 1 | TTAENEFVMLKK | | 1437.695 | M | 1437.728 | -0.033 | | 259 | 270 | | --- | --- | | 1 | RTTAENEFVMLK | | 1454.681 | M | 1454.686 | -0.005 | | 30 | 45 | | --- | --- | | 1 | TSFSSVSRSGGGGGGR | | 1484.718 | M | 1484.689 | 0.029 | | 347 | 358 | | --- | --- | | 1 | SRTEAESWYQTK | | 1514.729 | M | 1514.845 | -0.116 | | 194 | 206 | | --- | --- | | 1 | WALLQEQGTKTIK | | | |  | | --- | | | | **Input Summary** | | --- | | | Protein | 1  *    10   *    20   *    30   *    40   *    50      1 MSRQSSVSFRSGGSRSFSAASAITPSVSRTSFSSVSRSGGGGGGRISLGG    51 ACGAGGYGSRSLYNVGGSKRISYSSGGGSFRNQFGAGGFGFGGGAGSGFG   101 FGGGAGSGFGFGGGAGFGGGYGGAGFPVCPPGGIQEVTVNQNLLTPLNLQ   151 IDPTIQRVRTEEREQIKTLNNKFASFIDKVRFLEQQNKVLDTKWALLQEQ   201 GTKTIKQNLDPLFEQYINNLRRQLDGVLGERGRLDSELRNMQDLVEDYKN   251 KYEDEINKRTTAENEFVMLKKDVDAAYMNKVELEARVDALMDEINFMKMF   301 FDAELSQMQTHVSDTSVVLSMDNNRSLDLDSIIAEVKAQYEDIANRSRTE   351 AESWYQTKYEELQQTAGRHGDDLRNTKHEISEMNRMIQRLRSEIDNVKKQ   401 CANLQNAIAEAEQRGELALKDARNKLTELEEALQKAKQDMARLLREYQEL   451 MNTKLALDVEIATYRKLLEGEECRLSGEGVGPVNISVVTNSVSSGYGGGS   501 SIGVGSGFGGGLGSGFAGGLGPRFTRGGGGLGLGSGLSVGGSGFSAGSSQ   551 GGMSFGSGGGSGSSVKFVSTTSSSRRSFKS | | --- | --- | | | | Enzyme | | Name | Site(s) | Terminal | Mod. | Proline | Missed Cut | | --- | --- | --- | --- | --- | --- | | Trypsin | KR | C |  | N | 2 | | | --- | --- | --- | --- | --- | --- | --- | --- | --- | --- | --- | --- | --- | --- | | | | Global Modification | | Name | Site(s) | Formula | Min. Occ. | Max. Occ. | | --- | --- | --- | --- | --- | | Oxidation (M) | M | +O | Partial | | | | --- | --- | --- | --- | --- | --- | --- | --- | --- | --- | --- | --- | | | | Monoisotopic Masses  (+/- 100 ppm) | 1454.681 1081.590 905.456 1514.729 1437.695 1409.717 1025.522 1484.718 1241.572 1193.584 970.493 | | --- | --- | | | | Charge State | MH+ | | --- | --- | | | |
| | | [Name: (Q922U2) Keratin, type II cytoskeletal 5](http://prowl.rockefeller.edu/prowl/pmhelp.htm#PROTEIN_NAME) | | --- | | | --- | --- | | Number of Measured Peptides : 40  Number of Matched Peptides  : 2  Min. Coverage of Sequence   : 3% | | |  | | --- |  | Measured | Ave/ | Computed | Error | Residues | Missed |  | | --- | --- | --- | --- | --- | --- | --- | | Mass | Mono | Mass | (Da) | | Start | End | | --- | --- | | Cut | Peptide Sequence | | 947.487 | M | 947.438 | 0.049 | | 467 | 474 | | --- | --- | | 0 | LLEGEECR | | 1213.608 | M | 1213.677 | -0.069 | | 415 | 425 | | --- | --- | | 2 | GELALKDARNK | | | | **Unmatched Monoisotopic Masses:** 880.395 883.401 913.495 943.510 948.510 967.496 975.478 988.505 994.514 997.498 1011.504 1035.532 1061.519 1089.533 1092.536 1095.538 1106.541 1108.498 1110.576 1117.521 1127.553 1131.521 1143.511 1197.687 1202.632 1211.579 1224.616 1233.645 1304.614 1356.700 1380.667 1598.813 1637.841 1711.899 1789.879 1793.802 2112.999 2872.412 | | --- | | | | **Input Summary** | | --- | | | Protein | 1  *    10   *    20   *    30   *    40   *    50      1 MSRQSSVSFRSGGSRSFSAASAITPSVSRTSFSSVSRSGGGGGGRISLGG    51 ACGAGGYGSRSLYNVGGSKRISYSSGGGSFRNQFGAGGFGFGGGAGSGFG   101 FGGGAGSGFGFGGGAGFGGGYGGAGFPVCPPGGIQEVTVNQNLLTPLNLQ   151 IDPTIQRVRTEEREQIKTLNNKFASFIDKVRFLEQQNKVLDTKWALLQEQ   201 GTKTIKQNLDPLFEQYINNLRRQLDGVLGERGRLDSELRNMQDLVEDYKN   251 KYEDEINKRTTAENEFVMLKKDVDAAYMNKVELEARVDALMDEINFMKMF   301 FDAELSQMQTHVSDTSVVLSMDNNRSLDLDSIIAEVKAQYEDIANRSRTE   351 AESWYQTKYEELQQTAGRHGDDLRNTKHEISEMNRMIQRLRSEIDNVKKQ   401 CANLQNAIAEAEQRGELALKDARNKLTELEEALQKAKQDMARLLREYQEL   451 MNTKLALDVEIATYRKLLEGEECRLSGEGVGPVNISVVTNSVSSGYGGGS   501 SIGVGSGFGGGLGSGFAGGLGPRFTRGGGGLGLGSGLSVGGSGFSAGSSQ   551 GGMSFGSGGGSGSSVKFVSTTSSSRRSFKS | | --- | --- | | | | Enzyme | | Name | Site(s) | Terminal | Mod. | Proline | Missed Cut | | --- | --- | --- | --- | --- | --- | | Trypsin | KR | C |  | N | 2 | | | --- | --- | --- | --- | --- | --- | --- | --- | --- | --- | --- | --- | --- | --- | | | | Global Modification | | Name | Site(s) | Formula | Min. Occ. | Max. Occ. | | --- | --- | --- | --- | --- | | Oxidation (M) | M | +O | Partial | | | | --- | --- | --- | --- | --- | --- | --- | --- | --- | --- | --- | --- | | | | Monoisotopic Masses  (+/- 100 ppm) | 880.395 883.401 913.495 943.510 947.487 948.510 967.496 975.478 988.505 994.514 997.498 1011.504 1035.532 1061.519 1089.533 1092.536 1095.538 1106.541 1108.498 1110.576 1117.521 1127.553 1131.521 1143.511 1197.687 1202.632 1211.579 1213.608 1224.616 1233.645 1304.614 1356.700 1380.667 1598.813 1637.841 1711.899 1789.879 1793.802 2112.999 2872.412 | | --- | --- | | | | Charge State | MH+ | | --- | --- | | | |
| | | **[Name: (P17532) Tryptophan 5-hydroxylase 1](http://prowl.rockefeller.edu/prowl/pmhelp.htm#PROTEIN_NAME)** | | --- | | | --- | --- | | Number of Measured Peptides : 7  Number of Matched Peptides  : 5  Min. Coverage of Sequence   : 14% | | |  | | --- |  | Measured | Ave/ | Computed | Error | Residues | Missed |  | | --- | --- | --- | --- | --- | --- | --- | | Mass | Mono | Mass | (Da) | | Start | End | | --- | --- | | Cut | Peptide Sequence | | 1466.787 | M | 1466.751 | 0.036 | | 404 | 415 | | --- | --- | | 0 | YNPYTQSVQVLR | | 1620.701 | M | 1620.785 | -0.084 | | 102 | 114 | | --- | --- | | 1 | EDVMETVPWFPKK  (1)+O@M | | 1810.894 | M | 1810.921 | -0.027 | | 404 | 418 | | --- | --- | | 1 | YNPYTQSVQVLRDTK | | 1949.038 | M | 1949.048 | -0.010 | | 40 | 55 | | --- | --- | | 1 | IFQENHVSLLHIESRK | | 1993.081 | M | 1992.976 | 0.105 | | 150 | 166 | | --- | --- | | 1 | YFAELAMNYKHGDPIPK | | | | **Unmatched Monoisotopic Masses:** 2215.107 1208.590 | | --- | | | | **Input Summary** | | --- | | | Protein | 1  *    10   *    20   *    30   *    40   *    50      1 MIEDNKENKENKDHSSERGRVTLIFSLENEVGGLIKVLKIFQENHVSLLH    51 IESRKSKQRNSEFEIFVDCDISREQLNDIFPLLKSHATVLSVDSPDQLTA   101 KEDVMETVPWFPKKISDLDFCANRVLLYGSELDADHPGFKDNVYRRRRKY   151 FAELAMNYKHGDPIPKIEFTEEEIKTWGTIFRELNKLYPTHACREYLRNL   201 PLLSKYCGYREDNIPQLEDVSNFLKERTGFSIRPVAGYLSPRDFLSGLAF   251 RVFHCTQYVRHSSDPLYTPEPDTCHELLGHVPLLAEPSFAQFSQEIGLAS   301 LGASEETVQKLATCYFFTVEFGLCKQDGQLRVFGAGLLSSISELKHALSG   351 HAKVKPFDPKIACKQECLITSFQDVYFVSESFEDAKEKMREFAKTVKRPF   401 GLKYNPYTQSVQVLRDTKSITSAMNELRYDLDVISDALARVTRWPSV | | --- | --- | | | | Enzyme | | Name | Site(s) | Terminal | Mod. | Proline | Missed Cut | | --- | --- | --- | --- | --- | --- | | Trypsin | KR | C |  | N | 2 | | | --- | --- | --- | --- | --- | --- | --- | --- | --- | --- | --- | --- | --- | --- | | | | Global Modification | | Name | Site(s) | Formula | Min. Occ. | Max. Occ. | | --- | --- | --- | --- | --- | | Oxidation (M) | M | +O | Partial | | | | --- | --- | --- | --- | --- | --- | --- | --- | --- | --- | --- | --- | | | | Monoisotopic Masses  (+/- 100 ppm) | 1949.038 1620.701 1993.081 2215.107 1208.590 1466.787 1810.894 | | --- | --- | | | | Charge State | MH+ | | --- | --- | | | |
| | | **[Name: (Q8VCC6) Protein C20orf160 homolog](http://prowl.rockefeller.edu/prowl/pmhelp.htm#PROTEIN_NAME)** | | --- | | | --- | --- | | Number of Measured Peptides : 5  Number of Matched Peptides  : 4  Min. Coverage of Sequence   : 17% | | |  | | --- |  | Measured | Ave/ | Computed | Error | Residues | Missed |  | | --- | --- | --- | --- | --- | --- | --- | | Mass | Mono | Mass | (Da) | | Start | End | | --- | --- | | Cut | Peptide Sequence | | 881.433 | M | 881.446 | -0.013 | | 185 | 192 | | --- | --- | | 1 | APRPRNDG | | 913.463 | M | 913.421 | 0.042 | | 1 | 7 | | --- | --- | | 1 | MEYEAKK  (1)+O@M | | 1000.562 | M | 1000.518 | 0.044 | | 124 | 131 | | --- | --- | | 0 | DNEELILR | | 1107.614 | M | 1107.578 | 0.036 | | 27 | 36 | | --- | --- | | 1 | QAAFRSSVSR | | | | **Unmatched Monoisotopic Masses:** 960.549 | | --- | | | | **Input Summary** | | --- | | | Protein | 1  *    10   *    20   *    30   *    40   *    50      1 MEYEAKKGKKGFVSPIRRLVFPKAARQAAFRSSVSRRPLHSMPLYPPDYL    51 IDPHILLCDYLEKEVKFLGHLTWVTSSLNPSSRDELLQLLDTARQLKELP   101 LKTTPEQDSILSLSARCLLLTWRDNEELILRIPTHEIAAASYLQDDALHL   151 LVLKTGAARVWGWTLCLRAWMAAREVRVAIPALRAPRPRNDG | | --- | --- | | | | Enzyme | | Name | Site(s) | Terminal | Mod. | Proline | Missed Cut | | --- | --- | --- | --- | --- | --- | | Trypsin | KR | C |  | N | 2 | | | --- | --- | --- | --- | --- | --- | --- | --- | --- | --- | --- | --- | --- | --- | | | | Global Modification | | Name | Site(s) | Formula | Min. Occ. | Max. Occ. | | --- | --- | --- | --- | --- | | Oxidation (M) | M | +O | Partial | | | | --- | --- | --- | --- | --- | --- | --- | --- | --- | --- | --- | --- | | | | Monoisotopic Masses  (+/- 50 ppm) | 913.463 1107.614 960.549 1000.562 881.433 | | --- | --- | | | | Charge State | MH+ | | --- | --- | | | |
| | | **[Name: (P39054) Dynamin-2](http://prowl.rockefeller.edu/prowl/pmhelp.htm#PROTEIN_NAME)** | | --- | | | --- | --- | | Number of Measured Peptides : 9  Number of Matched Peptides  : 8  Min. Coverage of Sequence   : 13% | | |  | | --- |  | Measured | Ave/ | Computed | Error | Residues | Missed |  | | --- | --- | --- | --- | --- | --- | --- | | Mass | Mono | Mass | (Da) | | Start | End | | --- | --- | | Cut | Peptide Sequence | | 1224.672 | M | 1224.682 | -0.010 | | 189 | 199 | | --- | --- | | 1 | LAKEVDPQGLR | | 1257.678 | M | 1257.663 | 0.015 | | 5 | 15 | | --- | --- | | 0 | GMEELIPLVNK  (1)+O@M | | 1360.770 | M | 1360.688 | 0.082 | | 584 | 594 | | --- | --- | | 0 | HVFAIFNTEQR | | 1404.802 | M | 1404.743 | 0.059 | | 524 | 535 | | --- | --- | | 0 | GWLTINNISLMK  (1)+O@M | | 1536.875 | M | 1536.728 | 0.147 | | 267 | 279 | | --- | --- | | 1 | HMADRMGTPHLQK  (1)+O@M | | 1624.919 | M | 1624.914 | 0.005 | | 192 | 206 | | --- | --- | | 1 | EVDPQGLRTIGVITK | | 1624.919 | M | 1624.988 | -0.069 | | 224 | 237 | | --- | --- | | 2 | LLPLRRGYIGVVNR | | 1959.044 | M | 1958.965 | 0.079 | | 343 | 361 | | --- | --- | | 1 | RIEGSGDQVDTLELSGGAR | | 1980.926 | M | 1981.034 | -0.108 | | 326 | 342 | | --- | --- | | 1 | TKALLQMVQQFGVDFEK | | | | **Unmatched Monoisotopic Masses:** 1296.666 | | --- | | | | **Input Summary** | | --- | | | Protein | 1  *    10   *    20   *    30   *    40   *    50      1 MGNRGMEELIPLVNKLQDAFSSIGQSCHLDLPQIAVVGGQSAGKSSVLEN    51 FVGRDFLPRGSGIVTRRPLILQLIFSKTEYAEFLHCKSKKFTDFDEVRQE   101 IEAETDRVTGTNKGISPVPINLRVYSPHVLNLTLIDLPGITKVPVGDQPP   151 DIEYQIKDMILQFISRESSLILAVTPANMDLANSDALKLAKEVDPQGLRT   201 IGVITKLDLMDEGTDARDVLENKLLPLRRGYIGVVNRSQKDIEGKKDIRA   251 ALAAERKFFLSHPAYRHMADRMGTPHLQKTLNQQLTNHIRESLPTLRSKL   301 QSQLLSLEKEVEEYKNFRPDDPTRKTKALLQMVQQFGVDFEKRIEGSGDQ   351 VDTLELSGGARINRIFHERFPFELVKMEFDEKDLRREISYAIKNIHGVRT   401 GLFTPDLAFEAIVKKQVVKLKEPCLKCVDLVIQELISTVRQCTSKLSSYP   451 RLREETERIVTTYIREREGRTKDQILLLIDIEQSYINTNHEDFIGFANAQ   501 QRSTQLNKKRAIPNQGEILVIRRGWLTINNISLMKGGSKEYWFVLTAESL   551 SWYKDEEEKEKKYMLPLDNLKIRDVEKGFMSNKHVFAIFNTEQRNVYKDL   601 RQIELACDSQEDVDSWKASFLRAGVYPEKDQAENEDGAQENTFSMDPQLE   651 RQVETIRNLVDSYVAIINKSIRDLMPKTIMHLMINNTKAFIHHELLAYLY   701 SSADQSSLMEESAEQAQRRDDMLRMYHALKEALNIIGDISTSTVSTPVPP   751 PVDDTWLQNTSGHSPTPQRRPVSSVHPPGRPPAVRGPTPGPPLIPMPVGA   801 TSSFSAPPIPSRPGPQSVFANNDPFSAPPQIPSRPARIPPGIPPGVPSRR   851 APAAPSRPTIIRPAEPSLLD | | --- | --- | | | | Enzyme | | Name | Site(s) | Terminal | Mod. | Proline | Missed Cut | | --- | --- | --- | --- | --- | --- | | Trypsin | KR | C |  | N | 2 | | | --- | --- | --- | --- | --- | --- | --- | --- | --- | --- | --- | --- | --- | --- | | | | Global Modification | | Name | Site(s) | Formula | Min. Occ. | Max. Occ. | | --- | --- | --- | --- | --- | | Oxidation (M) | M | +O | Partial | | | | --- | --- | --- | --- | --- | --- | --- | --- | --- | --- | --- | --- | | | | Monoisotopic Masses  (+/- 100 ppm) | 1257.678 1296.666 1224.672 1624.919 1536.875 1980.926 1959.044 1404.802 1360.770 | | --- | --- | | | | Charge State | MH+ | | --- | --- | | | |
| | | **[Name: (P39054) Dynamin-2](http://prowl.rockefeller.edu/prowl/pmhelp.htm#PROTEIN_NAME)** | | --- | | | --- | --- | | Number of Measured Peptides : 11  Number of Matched Peptides  : 1  Min. Coverage of Sequence   : 1% | | |  | | --- |  | Measured | Ave/ | Computed | Error | Residues | Missed |  | | --- | --- | --- | --- | --- | --- | --- | | Mass | Mono | Mass | (Da) | | Start | End | | --- | --- | | Cut | Peptide Sequence | | 1454.802 | M | 1454.686 | 0.116 | | 78 | 89 | | --- | --- | | 1 | TEYAEFLHCKSK | | | | **Unmatched Monoisotopic Masses:** 869.429 1003.552 1060.557 1236.567 1448.817 1467.775 1492.849 1580.897 1712.985 2211.131 | | --- | | | | **Input Summary** | | --- | | | Protein | 1  *    10   *    20   *    30   *    40   *    50      1 MGNRGMEELIPLVNKLQDAFSSIGQSCHLDLPQIAVVGGQSAGKSSVLEN    51 FVGRDFLPRGSGIVTRRPLILQLIFSKTEYAEFLHCKSKKFTDFDEVRQE   101 IEAETDRVTGTNKGISPVPINLRVYSPHVLNLTLIDLPGITKVPVGDQPP   151 DIEYQIKDMILQFISRESSLILAVTPANMDLANSDALKLAKEVDPQGLRT   201 IGVITKLDLMDEGTDARDVLENKLLPLRRGYIGVVNRSQKDIEGKKDIRA   251 ALAAERKFFLSHPAYRHMADRMGTPHLQKTLNQQLTNHIRESLPTLRSKL   301 QSQLLSLEKEVEEYKNFRPDDPTRKTKALLQMVQQFGVDFEKRIEGSGDQ   351 VDTLELSGGARINRIFHERFPFELVKMEFDEKDLRREISYAIKNIHGVRT   401 GLFTPDLAFEAIVKKQVVKLKEPCLKCVDLVIQELISTVRQCTSKLSSYP   451 RLREETERIVTTYIREREGRTKDQILLLIDIEQSYINTNHEDFIGFANAQ   501 QRSTQLNKKRAIPNQGEILVIRRGWLTINNISLMKGGSKEYWFVLTAESL   551 SWYKDEEEKEKKYMLPLDNLKIRDVEKGFMSNKHVFAIFNTEQRNVYKDL   601 RQIELACDSQEDVDSWKASFLRAGVYPEKDQAENEDGAQENTFSMDPQLE   651 RQVETIRNLVDSYVAIINKSIRDLMPKTIMHLMINNTKAFIHHELLAYLY   701 SSADQSSLMEESAEQAQRRDDMLRMYHALKEALNIIGDISTSTVSTPVPP   751 PVDDTWLQNTSGHSPTPQRRPVSSVHPPGRPPAVRGPTPGPPLIPMPVGA   801 TSSFSAPPIPSRPGPQSVFANNDPFSAPPQIPSRPARIPPGIPPGVPSRR   851 APAAPSRPTIIRPAEPSLLD | | --- | --- | | | | Enzyme | | Name | Site(s) | Terminal | Mod. | Proline | Missed Cut | | --- | --- | --- | --- | --- | --- | | Trypsin | KR | C |  | N | 2 | | | --- | --- | --- | --- | --- | --- | --- | --- | --- | --- | --- | --- | --- | --- | | | | Global Modification | | Name | Site(s) | Formula | Min. Occ. | Max. Occ. | | --- | --- | --- | --- | --- | | Oxidation (M) | M | +O | Partial | | | | --- | --- | --- | --- | --- | --- | --- | --- | --- | --- | --- | --- | | | | Monoisotopic Masses  (+/- 100 ppm) | 869.429 1003.552 1060.557 1236.567 1448.817 1454.802 1467.775 1492.849 1580.897 1712.985 2211.131 | | --- | --- | | | | Charge State | MH+ | | --- | --- | | | |
| | | **[Name: (P52480) Pyruvate kinase, isozyme M2](http://prowl.rockefeller.edu/prowl/pmhelp.htm#PROTEIN_NAME)** | | --- | | | --- | --- | | Number of Measured Peptides : 12  Number of Matched Peptides  : 6  Min. Coverage of Sequence   : 15% | | |  | | --- |  | Measured | Ave/ | Computed | Error | Residues | Missed |  | | --- | --- | --- | --- | --- | --- | --- | | Mass | Mono | Mass | (Da) | | Start | End | | --- | --- | | Cut | Peptide Sequence | | 1108.506 | M | 1108.573 | -0.067 | | 270 | 278 | | --- | --- | | 1 | IENHEGVRR | | 1188.561 | M | 1188.628 | -0.067 | | 489 | 499 | | --- | --- | | 1 | VNLAMDVGKAR  (1)+O@M | | 1339.669 | M | 1339.722 | -0.053 | | 436 | 446 | | --- | --- | | 1 | SAHQVARYRPR | | 1836.934 | M | 1836.903 | 0.031 | | 278 | 293 | | --- | --- | | 1 | RFDEILEASDGIMVAR  (1)+O@M | | 2348.201 | M | 2348.258 | -0.057 | | 207 | 229 | | --- | --- | | 1 | GVNLPGAAVDLPAVSEKDIQ DLK | | 2787.152 | M | 2787.400 | -0.248 | | 279 | 304 | | --- | --- | | 1 | FDEILEASDGIMVARGDLGI EIPAEK | | | | **Unmatched Monoisotopic Masses:** 1358.694 2662.272 1036.506 1052.502 2509.140 2500.247 | | --- | | | | **Input Summary** | | --- | | | Protein | 1  *    10   *    20   *    30   *    40   *    50      1 PKPHSEAGTAFIQTQQLHAAMADTFLEHMCRLDIDSAPITARNTGIICTI    51 GPASRSVEMLKEMIKSGMNVARLNFSHGTHEYHAETIKNVREATESFASD   101 PILYRPVAVALDTKGPEIRTGLIKGSGTAEVELKKGATLKITLDNAYMEK   151 CDENILWLDYKNICKVVEVGSKIYVDDGLISLQVKEKGADFLVTEVENGG   201 SLGSKKGVNLPGAAVDLPAVSEKDIQDLKFGVEQDVDMVFASFIRKAADV   251 HEVRKVLGEKGKNIKIISKIENHEGVRRFDEILEASDGIMVARGDLGIEI   301 PAEKVFLAQKMMIGRCNRAGKPVICSTQMLEIMIKKPRPTRAEGSDVANA   351 VLDGADCIMLSGETAKGDYPLEAVRMQHLIAREAEAAIYHLQLFEELRRL   401 APITSDPTEAAAVGAVEASFKCCSGAIIVLTKSGRSAHQVARYRPRAPII   451 AVTRNPQTARQAHLYRGIFPVLCKDAVLNAWAEDVDLRVNLAMDVGKARG   501 FFKKGDVVIVLTGWRPGSGFTNTMRVVPVP | | --- | --- | | | | Enzyme | | Name | Site(s) | Terminal | Mod. | Proline | Missed Cut | | --- | --- | --- | --- | --- | --- | | Trypsin | KR | C |  | N | 2 | | | --- | --- | --- | --- | --- | --- | --- | --- | --- | --- | --- | --- | --- | --- | | | | Global Modification | | Name | Site(s) | Formula | Min. Occ. | Max. Occ. | | --- | --- | --- | --- | --- | | Oxidation (M) | M | +O | Partial | | | | --- | --- | --- | --- | --- | --- | --- | --- | --- | --- | --- | --- | | | | Monoisotopic Masses  (+/- 100 ppm) | 1358.694 2662.272 1108.506 1836.934 2787.152 1036.506 1052.502 2348.201 2509.140 1339.669 2500.247 1188.561 | | --- | --- | | | | Charge State | MH+ | | --- | --- | | | |
| | | **[Name: (P52480) Pyruvate kinase, isozyme M2](http://prowl.rockefeller.edu/prowl/pmhelp.htm#PROTEIN_NAME)** | | --- | | | --- | --- | | Number of Measured Peptides : 68  Number of Matched Peptides  : 4  Min. Coverage of Sequence   : 12% | | |  | | --- |  | Measured | Ave/ | Computed | Error | Residues | Missed |  | | --- | --- | --- | --- | --- | --- | --- | | Mass | Mono | Mass | (Da) | | Start | End | | --- | --- | | Cut | Peptide Sequence | | 1106.544 | M | 1106.582 | -0.038 | | 422 | 432 | | --- | --- | | 0 | CCSGAIIVLTK | | 1301.699 | M | 1301.676 | 0.023 | | 43 | 55 | | --- | --- | | 0 | NTGIICTIGPASR | | 1651.871 | M | 1651.886 | -0.015 | | 489 | 503 | | --- | --- | | 2 | VNLAMDVGKARGFFK | | 2417.224 | M | 2417.341 | -0.117 | | 166 | 187 | | --- | --- | | 2 | VVEVGSKIYVDDGLISLQVK EK | | | | **Unmatched Monoisotopic Masses:** 881.511 891.482 901.454 916.510 972.513 994.530 1032.508 1047.571 1064.526 1081.574 1164.642 1193.591 1200.666 1233.669 1253.628 1262.658 1264.629 1319.564 1335.687 1356.702 1380.648 1392.730 1433.751 1468.820 1522.772 1538.786 1637.831 1656.770 1699.838 1793.799 1795.979 1837.907 1850.906 1866.912 1940.947 1965.019 2022.037 2135.092 2149.058 2183.096 2249.053 2285.093 2328.153 2344.172 2357.139 2398.017 2565.263 2583.200 2642.330 2676.274 2690.298 2719.289 2747.327 2779.177 2871.426 2887.411 2901.440 2931.552 3051.669 3263.585 3275.603 3311.380 3322.823 3352.824 | | --- | | | | **Input Summary** | | --- | | | Protein | 1  *    10   *    20   *    30   *    40   *    50      1 PKPHSEAGTAFIQTQQLHAAMADTFLEHMCRLDIDSAPITARNTGIICTI    51 GPASRSVEMLKEMIKSGMNVARLNFSHGTHEYHAETIKNVREATESFASD   101 PILYRPVAVALDTKGPEIRTGLIKGSGTAEVELKKGATLKITLDNAYMEK   151 CDENILWLDYKNICKVVEVGSKIYVDDGLISLQVKEKGADFLVTEVENGG   201 SLGSKKGVNLPGAAVDLPAVSEKDIQDLKFGVEQDVDMVFASFIRKAADV   251 HEVRKVLGEKGKNIKIISKIENHEGVRRFDEILEASDGIMVARGDLGIEI   301 PAEKVFLAQKMMIGRCNRAGKPVICSTQMLEIMIKKPRPTRAEGSDVANA   351 VLDGADCIMLSGETAKGDYPLEAVRMQHLIAREAEAAIYHLQLFEELRRL   401 APITSDPTEAAAVGAVEASFKCCSGAIIVLTKSGRSAHQVARYRPRAPII   451 AVTRNPQTARQAHLYRGIFPVLCKDAVLNAWAEDVDLRVNLAMDVGKARG   501 FFKKGDVVIVLTGWRPGSGFTNTMRVVPVP | | --- | --- | | | | Enzyme | | Name | Site(s) | Terminal | Mod. | Proline | Missed Cut | | --- | --- | --- | --- | --- | --- | | Trypsin | KR | C |  | N | 2 | | | --- | --- | --- | --- | --- | --- | --- | --- | --- | --- | --- | --- | --- | --- | | | | Global Modification | | Name | Site(s) | Formula | Min. Occ. | Max. Occ. | | --- | --- | --- | --- | --- | | Oxidation (M) | M | +O | Partial | | | | --- | --- | --- | --- | --- | --- | --- | --- | --- | --- | --- | --- | | | | Monoisotopic Masses  (+/- 50 ppm) | 881.511 891.482 901.454 916.510 972.513 994.530 1032.508 1047.571 1064.526 1081.574 1106.544 1164.642 1193.591 1200.666 1233.669 1253.628 1262.658 1264.629 1301.699 1319.564 1335.687 1356.702 1380.648 1392.730 1433.751 1468.820 1522.772 1538.786 1637.831 1651.871 1656.770 1699.838 1793.799 1795.979 1837.907 1850.906 1866.912 1940.947 1965.019 2022.037 2135.092 2149.058 2183.096 2249.053 2285.093 2328.153 2344.172 2357.139 2398.017 2417.224 2565.263 2583.200 2642.330 2676.274 2690.298 2719.289 2747.327 2779.177 2871.426 2887.411 2901.440 2931.552 3051.669 3263.585 3275.603 3311.380 3322.823 3352.824 | | --- | --- | | | | Charge State | MH+ | | --- | --- | | | |

# Mascot Search Results

### Protein View

Match to: **ANGL6_MOUSE** Score: **58** Expect: **0.06**

**(Q8R0Z6) Angiopoietin-related protein 6 precursor (Angiopoietin-like 6) (Angiopoietin-related growt**

Nominal mass (M_r_): **51463**; Calculated pI value: **9.19**

NCBI BLAST search of [ANGL6_MOUSE](http://www.ncbi.nlm.nih.gov/blast/Blast.cgi?ALIGNMENTS=50&ALIGNMENT_VIEW=Pairwise&AUTO_FORMAT=Semiauto&CDD_SEARCH=on&CLIENT=web&COMPOSITION_BASED_STATISTICS=on&DATABASE=nr&DESCRIPTIONS=100&ENTREZ_QUERY=(none)&EXPECT=10&FILTER=L&FORMAT_BLOCK_ON_RESPAGE=None&FORMAT_OBJECT=Alignment&FORMAT_TYPE=HTML&GAPCOSTS=11+1&I_THRESH=0.001&LAYOUT=TwoWindows&MATRIX_NAME=BLOSUM62&NCBI_GI=on&PAGE=Proteins&PROGRAM=blastp&QUERY=MGTARLRKLQLLLLLGAWRALGGAARCRVTLVLSPQKATSAVCRSSEATQDSELATLRMRLGRHEELLRALQRRAAEGGALADEVRALREHSLTLNTRLGQLRAQLQQEARAEPDLGAEPAAALGLLAERALDAEAEARRTTARLQQLDAQLREHAQLMSQHSSLLGRLQRACAGPERGQQQVLPLPLAPLVPLSLVGSASNTSRRLDQTPEHQREQSLRQQGPPSSLLPTGHLAVPTRPVGPWRDCAEAHGAGHWQSGVYDLRLGRRVVAVWCEQQQEGGGWTVIQRRQDGSVNFFTNWQHYKAGFGRPEGEYWLGLEPVHQVTSRGDHELLILLEDWGGRAARAHYDSFSLEPESDHYRLRLGQYHGDAGDSLSWHNDKPFSTVDRDRDSYSGNCALYHRGGWWYHACAHSNLNGVWYHGGHYRSRYQDGVYWAEFRGGAYSLKKAVMLTRLVRL&SERVICE=plain&SET_DEFAULTS.x=9&SET_DEFAULTS.y=5&SHOW_OVERVIEW=on&WORD_SIZE=3&END_OF_HTTPGET=Yes) against nr

Unformatted [sequence string](http://mascot/mascot/cgi/getseq.pl?Sprot+ANGL6_MOUSE+seq) for pasting into other applications

Taxonomy: [Mus musculus](http://www.ncbi.nlm.nih.gov/htbin-post/Taxonomy/wgetorg?lvl=0&lin=f&id=10090)

Fixed modifications: Carbamidomethyl (C)

Variable modifications: Oxidation (M)

Cleavage by Trypsin: cuts C-term side of KR unless next residue is P

Number of mass values searched: **7**

Number of mass values matched: **4**

Sequence Coverage: **16%**

Matched peptides shown in **Bold Red**

**1** MGTARLRKLQ LLLLLGAWRA LGGAARCRVT LVLSPQKATS AVCR**SSEATQ**

**51 DSELATLRMR** LGRHEELLRA LQRRAAEGGA LADEVRALRE HSLTLNTRLG

**101** QLR**AQLQQEA RAEPDLGAEP AAALGLLAER** ALDAEAEARR TTARLQQLDA

**151** QLREHAQLMS QHSSLLGRLQ RACAGPERGQ QQVLPLPLAP LVPLSLVGSA

**201** SNTSRRLDQT PEHQREQSLR QQGPPSSLLP TGHLAVPTRP VGPWR**DCAEA**

**251 HGAGHWQSGV YDLR**LGRRVV AVWCEQQQEG GGWTVIQRR**Q DGSVNFFTNW**

**301 QHYK**AGFGRP EGEYWLGLEP VHQVTSRGDH ELLILLEDWG GRAARAHYDS

**351** FSLEPESDHY RLRLGQYHGD AGDSLSWHND KPFSTVDRDR DSYSGNCALY

**401** HRGGWWYHAC AHSNLNGVWY HGGHYRSRYQ DGVYWAEFRG GAYSLKKAVM

**451** LTRLVRL

  Residue Number  Increasing Mass  Decreasing Mass

**Start - End Observed Mr(expt) Mr(calc) Delta Miss Sequence**

**45 - 60 1794.7865 1793.7793 1793.8577 -0.0785 1 SSEATQDSELATLRMR**

**104 - 130 2788.5822 2787.5749 2787.4514 0.1235 1 AQLQQEARAEPDLGAEPAAALGLLAER**

**246 - 264 2128.9082 2127.9009 2127.9180 -0.0171 0 DCAEAHGAGHWQSGVYDLR**

**290 - 304 1870.9359 1869.9287 1869.8434 0.0853 0 QDGSVNFFTNWQHYK**

**No match to:** 1398.5865, 2749.2372, 3353.6773


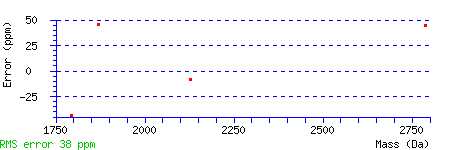


### Protein View

Match to: **ANXA2_MOUSE** Score: **82** Expect: **6.3e-005**

**(P07356) Annexin A2 (Annexin II) (Lipocortin II) (Calpactin I heavy chain) (Chromobindin-8) (p36) (**

Nominal mass (M_r_): **38806**; Calculated pI value: **7.53**

NCBI BLAST search of [ANXA2_MOUSE](http://www.ncbi.nlm.nih.gov/blast/Blast.cgi?ALIGNMENTS=50&ALIGNMENT_VIEW=Pairwise&AUTO_FORMAT=Semiauto&CDD_SEARCH=on&CLIENT=web&COMPOSITION_BASED_STATISTICS=on&DATABASE=nr&DESCRIPTIONS=100&ENTREZ_QUERY=(none)&EXPECT=10&FILTER=L&FORMAT_BLOCK_ON_RESPAGE=None&FORMAT_OBJECT=Alignment&FORMAT_TYPE=HTML&GAPCOSTS=11+1&I_THRESH=0.001&LAYOUT=TwoWindows&MATRIX_NAME=BLOSUM62&NCBI_GI=on&PAGE=Proteins&PROGRAM=blastp&QUERY=STVHEILCKLSLEGDHSTPPSAYGSVKPYTNFDAERDALNIETAVKTKGVDEVTIVNILTNRSNVQRQDIAFAYQRRTKKELPSALKSALSGHLETVILGLLKTPAQYDASELKASMKGLGTDEDSLIEIICSRTNQELQEINRVYKEMYKTDLEKDIISDTSGDFRKLMVALAKGRRAEDGSVIDYELIDQDARELYDAGVKRKGTDVPKWISIMTERSVCHLQKVFERYKSYSPYDMLESIKKEVKGDLENAFLNLVQCIQNKPLYFADRLYDSMKGKGTRDKVLIRIMVSRSEVDMLKIRSEFKRKYGKSLYYYIQQDTKGDYQKALLYLCGGDD&SERVICE=plain&SET_DEFAULTS.x=9&SET_DEFAULTS.y=5&SHOW_OVERVIEW=on&WORD_SIZE=3&END_OF_HTTPGET=Yes) against nr

Unformatted [sequence string](http://mascot/mascot/cgi/getseq.pl?Sprot+ANXA2_MOUSE+seq) for pasting into other applications

Taxonomy: [Mus musculus](http://www.ncbi.nlm.nih.gov/htbin-post/Taxonomy/wgetorg?lvl=0&lin=f&id=10090)

Fixed modifications: Carbamidomethyl (C)

Variable modifications: Oxidation (M)

Cleavage by Trypsin: cuts C-term side of KR unless next residue is P

Number of mass values searched: **6**

Number of mass values matched: **5**

Sequence Coverage: **18%**

Matched peptides shown in **Bold Red**

**1** STVHEILCKL SLEGDHSTPP SAYGSVKPYT NFDAERDALN IETAVKTK**GV**

**51 DEVTIVNILT NRSNVQRQDI AFAYQR**RTKK ELPSALK**SAL SGHLETVILG**

**101 LLK**TPAQYDA SELK**ASMKGL GTDEDSLIEI ICSR**TNQELQ EINRVYKEMY

**151** KTDLEKDIIS DTSGDFRKLM VALAKGRRAE DGSVIDYELI DQDARELYDA

**201** GVKRKGTDVP KWISIMTERS VCHLQKVFER YKSYSPYDML ESIKKEVKGD

**251** LENAFLNLVQ CIQNKPLYFA DRLYDSMKGK GTRDKVLIRI MVSRSEVDML

**301** KIRSEFKRKY GKSLYYYIQQ DTKGDYQKAL LYLCGGDD

  Residue Number  Increasing Mass  Decreasing Mass

**Start - End Observed Mr(expt) Mr(calc) Delta Miss Sequence**

**49 - 62 1542.9340 1541.9267 1541.8412 0.0855 0 GVDEVTIVNILTNR**

**63 - 76 1695.9370 1694.9297 1694.8488 0.0809 1 SNVQRQDIAFAYQR**

**88 - 103 1651.0530 1650.0457 1649.9715 0.0742 0 SALSGHLETVILGLLK**

**115 - 134 2210.9730 2209.9657 2210.0558 -0.0901 1 ASMKGLGTDEDSLIEIICSR**  Oxidation (M)

**119 - 134 1777.9270 1776.9197 1776.8563 0.0634 0 GLGTDEDSLIEIICSR**

**No match to:** 2939.5470


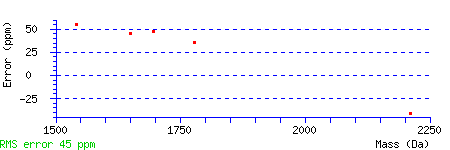


### Protein View

Match to: **C1RA_MOUSE** Score: **78** Expect: **0.00056**

**(Q8CG16) Complement C1r-A subcomponent precursor (EC 3.4.21.41) (Complement component 1, r-A subcom**

Nominal mass (M_r_): **81504**; Calculated pI value: **5.41**

NCBI BLAST search of [C1RA_MOUSE](http://www.ncbi.nlm.nih.gov/blast/Blast.cgi?ALIGNMENTS=50&ALIGNMENT_VIEW=Pairwise&AUTO_FORMAT=Semiauto&CDD_SEARCH=on&CLIENT=web&COMPOSITION_BASED_STATISTICS=on&DATABASE=nr&DESCRIPTIONS=100&ENTREZ_QUERY=(none)&EXPECT=10&FILTER=L&FORMAT_BLOCK_ON_RESPAGE=None&FORMAT_OBJECT=Alignment&FORMAT_TYPE=HTML&GAPCOSTS=11+1&I_THRESH=0.001&LAYOUT=TwoWindows&MATRIX_NAME=BLOSUM62&NCBI_GI=on&PAGE=Proteins&PROGRAM=blastp&QUERY=MWLFALLVTLFYGVEGSIYLPQKLYGEVTSPLYPKPYPSDLETTTVITVPMGYRVKLVFWQFDVEPSEGCFYDYVKISADKQTLGRFCGQLDSPLGNPPGSKEFMSQGNKMLLTFHTDFSNEENGTIMFYKGFLAYYQAVDLDECASQPNSVEEGLQPRCQHLCHNYVGGYFCSCHPGYELQKDGQSCQAECSSELYTEPSGYVSSLEYPQPYPPDLRCNYSIRVERGLTVHLKFLDPFEIDDHQQVHCPYDQLQIYANGKNLGEFCGKQRPPDLDTSSNAVDLLFFTDESGDSRGWKLHYTTETIKCPQPKALDEFTIIQDPQPQYQFRDYFIVTCKQGYQLMEGNQALLSFTAVCQNDGTWHRAMPRCKIKNCGQPQSLSNGDFRYITTKGVTTYEASIQYHCHEPYYKMLTRAGSSESMRGIYTCTAQGIWKNEEEGEKMPRCLPVCGKPVNPVTQKERIIRGQPARPGNFPWQAFTTTHGRGGGALLGDRWILTAAHTIYPKHHNKENDNANPKMLVFLGHTNVEQIKKLGHHPVRRVIIHPDYRQDEPNNFEGDIALLELENSVTLGPELLPICLPDNETFYGQGLMGYVSGFGITEDKLAFDLRFVRLPVADSEACQRWLQTKKDTSPFSQNMFCSGDPAVQQDACQGDSGGVFAVRDRNRDIWVATGIVSWGIGCGEGYGFYTKVLNYVDWIKKEMGDEN&SERVICE=plain&SET_DEFAULTS.x=9&SET_DEFAULTS.y=5&SHOW_OVERVIEW=on&WORD_SIZE=3&END_OF_HTTPGET=Yes) against nr

Unformatted [sequence string](http://mascot/mascot/cgi/getseq.pl?Sprot+C1RA_MOUSE+seq) for pasting into other applications

Taxonomy: [Mus musculus](http://www.ncbi.nlm.nih.gov/htbin-post/Taxonomy/wgetorg?lvl=0&lin=f&id=10090)

Fixed modifications: Carbamidomethyl (C)

Variable modifications: Oxidation (M)

Cleavage by Trypsin: cuts C-term side of KR unless next residue is P

Number of mass values searched: **19**

Number of mass values matched: **8**

Sequence Coverage: **17%**

Matched peptides shown in **Bold Red**

**1** MWLFALLVTL FYGVEGSIYL PQKLYGEVTS PLYPKPYPSD LETTTVITVP

**51** MGYRVKLVFW QFDVEPSEGC FYDYVKISAD KQTLGRFCGQ LDSPLGNPPG

**101** SKEFMSQGNK MLLTFHTDFS NEENGTIMFY K**GFLAYYQAV DLDECASQPN**

**151 SVEEGLQPR**C QHLCHNYVGG YFCSCHPGYE LQKDGQSCQA ECSSELYTEP

**201** SGYVSSLEYP QPYPPDLRCN YSIRVERGLT VHLKFLDPFE IDDHQQVHCP

**251** YDQLQIYANG KNLGEFCGKQ RPPDLDTSSN AVDLLFFTDE SGDSRGWKLH

**301** YTTETIKCPQ PK**ALDEFTII QDPQPQYQFR** DYFIVTCKQG YQLMEGNQAL

**351** LSFTAVCQND GTWHRAMPRC KIK**NCGQPQS LSNGDFR**YIT TK**GVTTYEAS**

**401 IQYHCHEPYY K**MLTRAGSSE SMRGIYTCTA QGIWKNEEEG EKMPR**CLPVC**

**451 GKPVNPVTQK** ERIIRGQPAR PGNFPWQAFT TTHGRGGGAL LGDRWILTAA

**501** HTIYPKHHNK ENDNANPKML VFLGHTNVEQ IKK**LGHHPVR RVIIHPDYR**Q

**551** DEPNNFEGDI ALLELENSVT LGPELLPICL PDNETFYGQG LMGYVSGFGI

**601** TEDKLAFDLR FVR**LPVADSE ACQR**WLQTKK DTSPFSQNMF CSGDPAVQQD

**651** ACQGDSGGVF AVRDRNRDIW VATGIVSWGI GCGEGYGFYT KVLNYVDWIK

**701** KEMGDEN

  Residue Number  Increasing Mass  Decreasing Mass

**Start - End Observed Mr(expt) Mr(calc) Delta Miss Sequence**

**132 - 159 3156.4930 3155.4857 3155.4505 0.0352 0 GFLAYYQAVDLDECASQPNSVEEGLQPR**

**313 - 330 2209.0858 2208.0785 2208.0851 -0.0066 0 ALDEFTIIQDPQPQYQFR**

**374 - 387 1579.6757 1578.6684 1578.6844 -0.0161 0 NCGQPQSLSNGDFR**

**393 - 411 2346.0511 2345.0438 2345.0422 0.0016 0 GVTTYEASIQYHCHEPYYK**

**446 - 460 1696.8257 1695.8184 1695.8800 -0.0615 0 CLPVCGKPVNPVTQK**

**534 - 541 971.5539 970.5467 970.5573 -0.0106 1 LGHHPVRR**

**542 - 549 1012.5438 1011.5365 1011.5501 -0.0136 0 VIIHPDYR**

**614 - 624 1245.5863 1244.5790 1244.5819 -0.0029 0 LPVADSEACQR**

**No match to:** 1189.5754, 1320.5889, 1330.7532, 1479.7654, 1497.7481, 1599.7701, 1909.9380, 2227.0810, 2231.0790, 2233.0763, 2247.0757


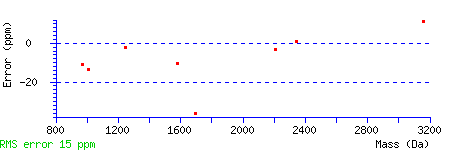


### Protein View

Match to: **CS1A_MOUSE** Score: **66** Expect: **0.0023**

**(Q8CG14) Complement C1s-A subcomponent precursor (EC 3.4.21.42) (C1 esterase) [Contains: Complement**

Nominal mass (M_r_): **78347**; Calculated pI value: **4.97**

NCBI BLAST search of [CS1A_MOUSE](http://www.ncbi.nlm.nih.gov/blast/Blast.cgi?ALIGNMENTS=50&ALIGNMENT_VIEW=Pairwise&AUTO_FORMAT=Semiauto&CDD_SEARCH=on&CLIENT=web&COMPOSITION_BASED_STATISTICS=on&DATABASE=nr&DESCRIPTIONS=100&ENTREZ_QUERY=(none)&EXPECT=10&FILTER=L&FORMAT_BLOCK_ON_RESPAGE=None&FORMAT_OBJECT=Alignment&FORMAT_TYPE=HTML&GAPCOSTS=11+1&I_THRESH=0.001&LAYOUT=TwoWindows&MATRIX_NAME=BLOSUM62&NCBI_GI=on&PAGE=Proteins&PROGRAM=blastp&QUERY=MWCLVLFSLLASFSAEPTMHGEILSPNYPQAYPNDVVKSWDIEVPEGFGIHLYFTHVDIEPSESCAYDSVQIISGGIEEGRLCGQKTSKSPNSPIIEEFQFPYNKLQVVFTSDFSNEERFTGFAAYYTAIDINECTDFTDVPCSHFCNNFIGGYFCSCPPEYFLHDDMRNCGVNCSGDVFTALIGEISSPNYPNPYPENSRCEYQIQLQEGFQVVVTMQREDFDVEPADSEGNCPDSLTFASKNQQFGPYCGNGFPGPLTIRTQSNTLGIVFQTDLMGQKKGWKLRYHGDPISCAKKITANSTWEPDKAKYVFKDVVKITCVDGFEVVEGHVSSTSYYSTCQSDGQWSNSGLKCQPVYCGIPDPIANGKVEEPENSVFGTVVHYTCEEPYYYMEHEEGGEYRCAANGRWVNDQLGIELPRCIPACGVPTEPFQVHQRIFGGQPAKIENFPWQVFFNHPRASGALINEYWVLTAAHVLEKISDPLMYVGTMSVRTTLLENAQRLYSKRVFIHPSWKKEDDPNTRTNFDNDIALVQLKDPVKMGPKVSPICLPGTSSEYNVSPGDMGLISGWGSTEKKVFVINLRGAKVPVTSLETCKQVKEENPTVRPEDYVFTDNMICAGEKGVDSCHGDSGGAFAFQVPNVTVPKFYVAGLVSWGKRCGTYGVYTKVKNYVDWILKTMQENSGPRKD&SERVICE=plain&SET_DEFAULTS.x=9&SET_DEFAULTS.y=5&SHOW_OVERVIEW=on&WORD_SIZE=3&END_OF_HTTPGET=Yes) against nr

Unformatted [sequence string](http://mascot/mascot/cgi/getseq.pl?Sprot+CS1A_MOUSE+seq) for pasting into other applications

Taxonomy: [Mus musculus](http://www.ncbi.nlm.nih.gov/htbin-post/Taxonomy/wgetorg?lvl=0&lin=f&id=10090)

Fixed modifications: Carbamidomethyl (C)

Variable modifications: Oxidation (M)

Cleavage by Trypsin: cuts C-term side of KR unless next residue is P

Number of mass values searched: **13**

Number of mass values matched: **6**

Sequence Coverage: **15%**

Matched peptides shown in **Bold Red**

**1** MWCLVLFSLL ASFSAEPTMH GEILSPNYPQ AYPNDVVKSW DIEVPEGFGI

**51** HLYFTHVDIE PSESCAYDSV QIISGGIEEG RLCGQKTSK**S PNSPIIEEFQ**

**101 FPYNKLQVVF TSDFSNEER**F TGFAAYYTAI DINECTDFTD VPCSHFCNNF

**151** IGGYFCSCPP EYFLHDDMRN CGVNCSGDVF TALIGEISSP NYPNPYPENS

**201** RCEYQIQLQE GFQVVVTMQR EDFDVEPADS EGNCPDSLTF ASKNQQFGPY

**251** CGNGFPGPLT IR**TQSNTLGI VFQTDLMGQK** KGWKLRYHGD PISCAKKITA

**301** NSTWEPDKAK YVFKDVVKIT CVDGFEVVEG HVSSTSYYST CQSDGQWSNS

**351** GLKCQPVYCG IPDPIANGKV EEPENSVFGT VVHYTCEEPY YYMEHEEGGE

**401** YRCAANGR**WV NDQLGIELPR** CIPACGVPTE PFQVHQRIFG GQPAK**IENFP**

**451 WQVFFNHPR**A SGALINEYWV LTAAHVLEKI SDPLMYVGTM SVRTTLLENA

**501** QRLYSKRVFI HPSWKKEDDP NTRTNFDNDI ALVQLKDPVK MGPK**VSPICL**

**551 PGTSSEYNVS PGDMGLISGW GSTEKK**VFVI NLRGAKVPVT SLETCKQVKE

**601** ENPTVRPEDY VFTDNMICAG EKGVDSCHGD SGGAFAFQVP NVTVPKFYVA

**651** GLVSWGKRCG TYGVYTKVKN YVDWILKTMQ ENSGPRKD

  Residue Number  Increasing Mass  Decreasing Mass

**Start - End Observed Mr(expt) Mr(calc) Delta Miss Sequence**

**90 - 105 1909.9267 1908.9194 1908.9257 -0.0063 0 SPNSPIIEEFQFPYNK**

**106 - 119 1670.8318 1669.8245 1669.7947 0.0298 0 LQVVFTSDFSNEER**

**263 - 280 1996.9936 1995.9864 1995.9935 -0.0071 0 TQSNTLGIVFQTDLMGQK**  Oxidation (M)

**409 - 420 1439.7729 1438.7656 1438.7568 0.0088 0 WVNDQLGIELPR**

**446 - 459 1830.9043 1829.8970 1829.9001 -0.0031 0 IENFPWQVFFNHPR**

**545 - 576 3353.6705 3352.6632 3352.5954 0.0678 1 VSPICLPGTSSEYNVSPGDMGLISGWGSTEKK**

**No match to:** 905.6560, 1479.8138, 1609.8039, 1794.8308, 2124.0165, 2314.1454, 3339.6596


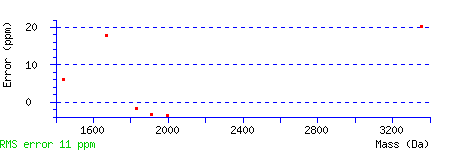


### Protein View

Match to: **LAC1_MOUSE** Score: **70** Expect: **0.00095**

**(P01843) Ig lambda-1 chain C region**

Nominal mass (M_r_): **11739**; Calculated pI value: **5.87**

NCBI BLAST search of [LAC1_MOUSE](http://www.ncbi.nlm.nih.gov/blast/Blast.cgi?ALIGNMENTS=50&ALIGNMENT_VIEW=Pairwise&AUTO_FORMAT=Semiauto&CDD_SEARCH=on&CLIENT=web&COMPOSITION_BASED_STATISTICS=on&DATABASE=nr&DESCRIPTIONS=100&ENTREZ_QUERY=(none)&EXPECT=10&FILTER=L&FORMAT_BLOCK_ON_RESPAGE=None&FORMAT_OBJECT=Alignment&FORMAT_TYPE=HTML&GAPCOSTS=11+1&I_THRESH=0.001&LAYOUT=TwoWindows&MATRIX_NAME=BLOSUM62&NCBI_GI=on&PAGE=Proteins&PROGRAM=blastp&QUERY=QPKSSPSVTLFPPSSEELETNKATLVCTITDFYPGVVTVDWKVDGTPVTQGMETTQPSKQSNNKYMASSYLTLTARAWERHSSYSCQVTHEGHTVEKSLSRADCS&SERVICE=plain&SET_DEFAULTS.x=9&SET_DEFAULTS.y=5&SHOW_OVERVIEW=on&WORD_SIZE=3&END_OF_HTTPGET=Yes) against nr

Unformatted [sequence string](http://mascot/mascot/cgi/getseq.pl?Sprot+LAC1_MOUSE+seq) for pasting into other applications

Taxonomy: [Mus musculus](http://www.ncbi.nlm.nih.gov/htbin-post/Taxonomy/wgetorg?lvl=0&lin=f&id=10090)

Fixed modifications: Carbamidomethyl (C)

Variable modifications: Oxidation (M)

Cleavage by Trypsin: cuts C-term side of KR unless next residue is P

Number of mass values searched: **10**

Number of mass values matched: **4**

Sequence Coverage: **61%**

Matched peptides shown in **Bold Red**

**1** QPK**SSPSVTL FPPSSEELET NK**ATLVCTIT DFYPGVVTVD WK**VDGTPVTQ**

**51 GMETTQPSK**Q SNNK**YMASSY LTLTAR**AWER **HSSYSCQVTH EGHTVEK**SLS

**101** RADCS

  Residue Number  Increasing Mass  Decreasing Mass

**Start - End Observed Mr(expt) Mr(calc) Delta Miss Sequence**

**4 - 22 2048.9977 2047.9904 2047.9949 -0.0045 0 SSPSVTLFPPSSEELETNK**

**43 - 59 1791.8340 1790.8267 1790.8356 -0.0089 0 VDGTPVTQGMETTQPSK**  Oxidation (M)

**65 - 76 1392.6876 1391.6803 1391.6755 0.0049 0 YMASSYLTLTAR**  Oxidation (M)

**81 - 97 1985.8900 1984.8827 1984.8697 0.0130 0 HSSYSCQVTHEGHTVEK**

**No match to:** 882.4645, 923.4523, 956.4942, 1265.6536, 1347.5860, 2432.1746


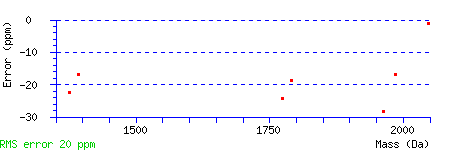


### Protein View

Match to: **ACTG_MOUSE** Score: **188** Expect: **1.6e-015**

**(P63260) Actin, cytoplasmic 2 (Gamma-actin)**

Nominal mass (M_r_): **42108**; Calculated pI value: **5.31**

NCBI BLAST search of [ACTG_MOUSE](http://www.ncbi.nlm.nih.gov/blast/Blast.cgi?ALIGNMENTS=50&ALIGNMENT_VIEW=Pairwise&AUTO_FORMAT=Semiauto&CDD_SEARCH=on&CLIENT=web&COMPOSITION_BASED_STATISTICS=on&DATABASE=nr&DESCRIPTIONS=100&ENTREZ_QUERY=(none)&EXPECT=10&FILTER=L&FORMAT_BLOCK_ON_RESPAGE=None&FORMAT_OBJECT=Alignment&FORMAT_TYPE=HTML&GAPCOSTS=11+1&I_THRESH=0.001&LAYOUT=TwoWindows&MATRIX_NAME=BLOSUM62&NCBI_GI=on&PAGE=Proteins&PROGRAM=blastp&QUERY=MEEEIAALVIDNGSGMCKAGFAGDDAPRAVFPSIVGRPRHQGVMVGMGQKDSYVGDEAQSKRGILTLKYPIEHGIVTNWDDMEKIWHHTFYNELRVAPEEHPVLLTEAPLNPKANREKMTQIMFETFNTPAMYVAIQAVLSLYASGRTTGIVMDSGDGVTHTVPIYEGYALPHAILRLDLAGRDLTDYLMKILTERGYSFTTTAEREIVRDIKEKLCYVALDFEQEMATAASSSSLEKSYELPDGQVITIGNERFRCPEALFQPSFLGMESCGIHETTFNSIMKCDVDIRKDLYANTVLSGGTTMYPGIADRMQKEITALAPSTMKIKIIAPPERKYSVWIGGSILASLSTFQQMWISKQEYDESGPSIVHRKCF&SERVICE=plain&SET_DEFAULTS.x=9&SET_DEFAULTS.y=5&SHOW_OVERVIEW=on&WORD_SIZE=3&END_OF_HTTPGET=Yes) against nr

Unformatted [sequence string](http://mascot/mascot/cgi/getseq.pl?Sprot+ACTG_MOUSE+seq) for pasting into other applications

Taxonomy: [Mus musculus](http://www.ncbi.nlm.nih.gov/htbin-post/Taxonomy/wgetorg?lvl=0&lin=f&id=10090)

Fixed modifications: Carbamidomethyl (C)

Variable modifications: Oxidation (M)

Cleavage by Trypsin: cuts C-term side of KR unless next residue is P

Number of mass values searched: **33**

Number of mass values matched: **16**

Sequence Coverage: **53%**

Matched peptides shown in **Bold Red**

**1** **MEEEIAALVI DNGSGMCKAG FAGDDAPRAV FPSIVGRPR**H QGVMVGMGQK

**51** **DSYVGDEAQS KR**GILTLKYP IEHGIVTNWD DMEKIWHHTF YNELR**VAPEE**

**101 HPVLLTEAPL NPK**ANREKMT QIMFETFNTP AMYVAIQAVL SLYASGR**TTG**

**151 IVMDSGDGVT HTVPIYEGYA LPHAILRLDL AGRDLTDYLM K**ILTER**GYSF**

**201 TTTAEREIVR** DIKEKLCYVA LDFEQEMATA ASSSSLEK**SY ELPDGQVITI**

**251 GNER**FRCPEA LFQPSFLGME SCGIHETTFN SIMKCDVDIR K**DLYANTVLS**

**301 GGTTMYPGIA DRMQKEITAL APSTMKIKII APPERK**YSVW IGGSILASLS

**351** TFQQMWISK**Q EYDESGPSIV HRK**CF

  Residue Number  Increasing Mass  Decreasing Mass

**Start - End Observed Mr(expt) Mr(calc) Delta Miss Sequence**

**1 - 18 1999.0110 1998.0037 1997.8743 0.1294 0 MEEEIAALVIDNGSGMCK**  2 Oxidation (M)

**19 - 28 976.4206 975.4133 975.4409 -0.0276 0 AGFAGDDAPR**

**29 - 39 1198.6750 1197.6677 1197.6982 -0.0304 0 AVFPSIVGRPR**

**51 - 62 1354.5910 1353.5837 1353.6160 -0.0323 1 DSYVGDEAQSKR**

**96 - 113 1954.0160 1953.0087 1953.0571 -0.0483 0 VAPEEHPVLLTEAPLNPK**

**148 - 177 3199.5310 3198.5237 3198.6019 -0.0781 0 TTGIVMDSGDGVTHTVPIYEGYALPHAILR**  Oxidation (M)

**178 - 191 1639.8020 1638.7947 1638.8287 -0.0339 1 LDLAGRDLTDYLMK**  Oxidation (M)

**197 - 206 1132.4910 1131.4837 1131.5196 -0.0359 0 GYSFTTTAER**

**197 - 210 1629.7810 1628.7737 1628.8158 -0.0420 1 GYSFTTTAEREIVR**

**239 - 254 1790.8580 1789.8507 1789.8846 -0.0338 0 SYELPDGQVITIGNER**

**292 - 312 2231.0020 2229.9947 2230.0575 -0.0628 0 DLYANTVLSGGTTMYPGIADR**  Oxidation (M)

**313 - 326 1548.8100 1547.8027 1547.8051 -0.0024 1 MQKEITALAPSTMK**

**327 - 335 1036.6250 1035.6177 1035.6440 -0.0263 1 IKIIAPPER**

**329 - 336 923.5398 922.5325 922.5600 -0.0274 1 IIAPPERK**

**360 - 372 1516.6650 1515.6577 1515.6953 -0.0376 0 QEYDESGPSIVHR**

**360 - 373 1644.7630 1643.7557 1643.7903 -0.0346 1 QEYDESGPSIVHRK**

**No match to:** 870.4225, 1014.3740, 1022.0900, 1060.0340, 1131.6020, 1164.7210, 1231.6450, 1274.5750, 1409.6560, 1440.6980, 1443.7210, 1461.7110, 1624.8050, 1807.9170, 1829.7860, 1838.7430, 1992.9700


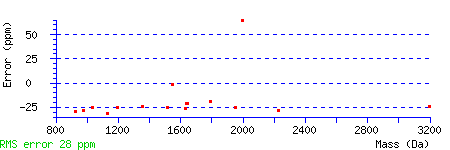
Formularende

### Protein View

Match to: **CUL1_MOUSE** Score: **62** Expect: **0.0069**

**(Q9WTX6) Cullin-1 (CUL-1)**

Nominal mass (M_r_): **90319**; Calculated pI value: **8.19**

NCBI BLAST search of [CUL1_MOUSE](http://www.ncbi.nlm.nih.gov/blast/Blast.cgi?ALIGNMENTS=50&ALIGNMENT_VIEW=Pairwise&AUTO_FORMAT=Semiauto&CDD_SEARCH=on&CLIENT=web&COMPOSITION_BASED_STATISTICS=on&DATABASE=nr&DESCRIPTIONS=100&ENTREZ_QUERY=(none)&EXPECT=10&FILTER=L&FORMAT_BLOCK_ON_RESPAGE=None&FORMAT_OBJECT=Alignment&FORMAT_TYPE=HTML&GAPCOSTS=11+1&I_THRESH=0.001&LAYOUT=TwoWindows&MATRIX_NAME=BLOSUM62&NCBI_GI=on&PAGE=Proteins&PROGRAM=blastp&QUERY=MSSNRSQNPHGLKQIGLDQIWDDLRAGIQQVYTRQSMAKSRYMELYTHVYNYCTSVHQSNQARGAGVPPSKSKKGQTPGGAQFVGLELYKRLKEFLKNYLTNLLKDGEDLMDESVLKFYTQQWEDYRFSSKVLNGICAYLNRHWVRRECDEGRKGIYEIYSLALVTWRDCLFRPLNKQVTNAVLKLIEKERNGETINTRLISGVVQSYVELGLNEDDAFAKGPTLTVYKESFESQFLADTERFYTRESTEFLQQNPVTEYMKKAEARLLEEQRRVQVYLHESTQDELARKCEQVLIEKHLEIFHTEFQNLLDADKNEDLGRMYNLVSRIQDGLGELKKLLETHIHNQGLAAIEKCGEAALNDPKMYVQTVLDVHKKYNALVMSAFNNDAGFVAALDKACGRFINNNAVTKMAQSSSKSPELLARYCDSLLKKSSKNPEEAELEDTLNQVMVVFKYIEDKDVFQKFYAKMLAKRLVHQNSASDDAEASMISKLKQACGFEYTSKLQRMFQDIGVSKDLNEQFKKHLTNSEPLDLDFSIQVLSSGSWPFQQSCTFALPSELERSYQRFTAFYASRHSGRKLTWLYQLSKGELVTNCFKNRYTLQASTFQMAILLQYNTEDAYTVQQLTDSTQIKMDILAQVLQILLKSKLLVLEDENANVDEVELKPDTLIKLYLGYKNKKLRVNINVPMKTEQKQEQETTHKNIEEDRKLLIQAAIVRIMKMRKVLKHQQLLGEVLTQLSSRFKPRVPVIKKCIDILIEKEYLERVDGEKDTYSYLA&SERVICE=plain&SET_DEFAULTS.x=9&SET_DEFAULTS.y=5&SHOW_OVERVIEW=on&WORD_SIZE=3&END_OF_HTTPGET=Yes) against nr

Unformatted [sequence string](http://mascot/mascot/cgi/getseq.pl?Sprot+CUL1_MOUSE+seq) for pasting into other applications

Taxonomy: [Mus musculus](http://www.ncbi.nlm.nih.gov/htbin-post/Taxonomy/wgetorg?lvl=0&lin=f&id=10090)

Fixed modifications: Carbamidomethyl (C)

Variable modifications: Oxidation (M)

Cleavage by Trypsin: cuts C-term side of KR unless next residue is P

Number of mass values searched: **18**

Number of mass values matched: **7**

Sequence Coverage: **15%**

Matched peptides shown in **Bold Red**

**1** MSSNRSQNPH GLKQIGLDQI WDDLRAGIQQ VYTRQSMAKS RYMELYTHVY

**51** NYCTSVHQSN QARGAGVPPS KSK**KGQTPGG AQFVGLELYK** RLKEFLKNYL

**101** TNLLKDGEDL MDESVLKFYT QQWEDYRFSS KVLNGICAYL NRHWVRRECD

**151** EGRKGIYEIY SLALVTWRDC LFRPLNKQVT NAVLKLIEKE RNGETINTRL

**201** ISGVVQSYVE LGLNEDDAFA KGPTLTVYKE SFESQFLADT ERFYTRESTE

**251** FLQQNPVTEY MKKAEARLLE EQRRVQVYLH ESTQDELARK CEQVLIEKHL

**301** EIFHTEFQNL LDADK**NEDLG RMYNLVSR**IQ DGLGELKKLL ETHIHNQGLA

**351** AIEKCGEAAL NDPKMYVQTV LDVHKK**YNAL VMSAFNNDAG FVAALDK**ACG

**401** RFINNNAVTK MAQSSSKSPE LLARYCDSLL KKSSKNPEEA ELEDTLNQVM

**451** VVFKYIEDKD VFQKFYAKML AKRLVHQNSA SDDAEASMIS KLKQACGFEY

**501** TSKLQRMFQD IGVSKDLNEQ FKKHLTNSEP LDLDFSIQVL SSGSWPFQQS

**551** CTFALPSELE RSYQRFTAFY ASRHSGRKLT WLYQLSK**GEL VTNCFKNR**YT

**601** LQASTFQMAI LLQYNTEDAY TVQQLTDSTQ IKMDILAQVL QILLKSK**LLV**

**651 LEDENANVDE VELKPDTLIK LYLGYK**NKKL RVNINVPMKT EQK**QEQETTH**

**701 KNIEEDR**KLL IQAAIVRIMK MRKVLK**HQQL LGEVLTQLSS R**FKPRVPVIK

**751** KCIDILIEKE YLERVDGEKD TYSYLA

  Residue Number  Increasing Mass  Decreasing Mass

**Start - End Observed Mr(expt) Mr(calc) Delta Miss Sequence**

**74 - 90 1792.8230 1791.8157 1791.9518 -0.1361 1 KGQTPGGAQFVGLELYK**

**316 - 328 1582.8729 1581.8657 1581.7569 0.1088 1 NEDLGRMYNLVSR**  Oxidation (M)

**377 - 397 2247.1634 2246.1561 2246.0677 0.0885 0 YNALVMSAFNNDAGFVAALDK**  Oxidation (M)

**588 - 598 1337.7287 1336.7214 1336.6557 0.0657 1 GELVTNCFKNR**

**648 - 676 3346.6390 3345.6317 3345.7958 -0.1641 1 LLVLEDENANVDEVELKPDTLIKLYLGYK**

**694 - 707 1756.7824 1755.7751 1755.8023 -0.0272 1 QEQETTHKNIEEDR**

**727 - 741 1708.9529 1707.9456 1707.9267 0.0189 0 HQQLLGEVLTQLSSR**

**No match to:** 907.7674, 1060.0550, 1060.5448, 1066.0873, 1082.0595, 1277.1220, 1468.8027, 1669.8706, 2226.1242, 2234.0743, 2254.1625


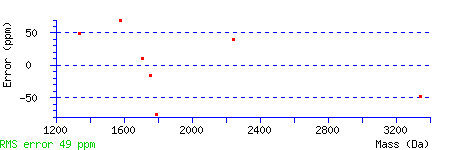


### Protein View

**Match to OST5_MOUSE** Score: **70** Expect: **0.00097**

**(Q8BSL4) Heparan sulfate glucosamine 3-O-sulfotransferase 5 (EC 2.8.2.23) (Heparan sulfate D-glucos**

Nominal mass (M_r_): **40674**; Calculated pI value: **9.72**

NCBI BLAST search of [OST5_MOUSE](http://www.ncbi.nlm.nih.gov/blast/Blast.cgi?ALIGNMENTS=50&ALIGNMENT_VIEW=Pairwise&AUTO_FORMAT=Semiauto&CDD_SEARCH=on&CLIENT=web&COMPOSITION_BASED_STATISTICS=on&DATABASE=nr&DESCRIPTIONS=100&ENTREZ_QUERY=(none)&EXPECT=10&FILTER=L&FORMAT_BLOCK_ON_RESPAGE=None&FORMAT_OBJECT=Alignment&FORMAT_TYPE=HTML&GAPCOSTS=11+1&I_THRESH=0.001&LAYOUT=TwoWindows&MATRIX_NAME=BLOSUM62&NCBI_GI=on&PAGE=Proteins&PROGRAM=blastp&QUERY=MLFKQQVWLRQKLLVLGSLAVGSLLYLVARVGSLDRLQPICPVESRFGGAHNQAELPLRALQFKRGLLHEFRKGNSSKEQVHLHDLVQQLPKAIIIGVRKGGTRALLEMLNLHPAVVKASQEIHFFDNDENYAKGIEWYRKKMPFSYPQQITIEKSPAYFITEEVPERIYKMNSSIKLLIIVREPTTRAISDYTQVLEGKERKNKTYYKFEKLAIDPNTCEVNTKYKAVRTSIYTKHLERWLKYFPIEQFHIVDGDRLITEPLPELQLVEKFLNLPPRISQYNLYFNATRGFYCLRFNIIFNKCLAGSKGRIHPEVDPSVITKLRKFFHPFNQKFYQITGRTLNWP&SERVICE=plain&SET_DEFAULTS.x=9&SET_DEFAULTS.y=5&SHOW_OVERVIEW=on&WORD_SIZE=3&END_OF_HTTPGET=Yes) against nr

Unformatted [sequence string](http://mascot/mascot/cgi/getseq.pl?Sprot+OST5_MOUSE+seq) for pasting into other applications

Taxonomy: [Mus musculus](http://www.ncbi.nlm.nih.gov/htbin-post/Taxonomy/wgetorg?lvl=0&lin=f&id=10090)

Fixed modifications: Carbamidomethyl (C)

Variable modifications: Oxidation (M)

Cleavage by Trypsin: cuts C-term side of KR unless next residue is P

Number of mass values searched: **13**

Number of mass values matched: **6**

Sequence Coverage: **23%**

Matched peptides shown in **Bold Red**

**1** MLFKQQVWLR QKLLVLGSLA VGSLLYLVAR VGSLDRLQPI CPVESR**FGGA**

**51 HNQAELPLR**A LQFKRGLLHE FRKGNSSKEQ VHLHDLVQQL PKAIIIGVRK

**101** GGTRALLEML NLHPAVVKAS QEIHFFDNDE NYAKGIEWYR K**KMPFSYPQQ**

**151 ITIEK**SPAYF ITEEVPERIY KMNSSIKLLI IVREPTTRAI SDYTQVLEGK

**201** ERKNKTYYKF EK**LAIDPNTC EVNTKYK**AVR **TSIYTKHLER WLKYFPIEQF**

**251 HIVDGDR**LIT EPLPELQLVE KFLNLPPRIS QYNLYFNATR GFYCLRFNII

**301** FNKCLAGSKG R**IHPEVDPSV ITKLR**KFFHP FNQKFYQITG RTLNWP

  Residue Number  Increasing Mass  Decreasing Mass

**Start - End Observed Mr(expt) Mr(calc) Delta Miss Sequence**

**47 - 59 1409.7810 1408.7737 1408.7211 0.0527 0 FGGAHNQAELPLR**

**142 - 155 1709.9610 1708.9537 1708.8858 0.0679 1 KMPFSYPQQITIEK**

**213 - 227 1765.9760 1764.9687 1764.8716 0.0971 1 LAIDPNTCEVNTKYK**

**231 - 240 1247.6730 1246.6657 1246.6669 -0.0012 1 TSIYTKHLER**

**241 - 257 2163.0190 2162.0117 2162.0948 -0.0831 1 WLKYFPIEQFHIVDGDR**

**312 - 325 1603.9150 1602.9077 1602.9093 -0.0016 1 IHPEVDPSVITKLR**

**No match to:** 910.6852, 1529.8980, 1619.9160, 1910.0050, 2269.1230, 2347.0660, 2978.7580


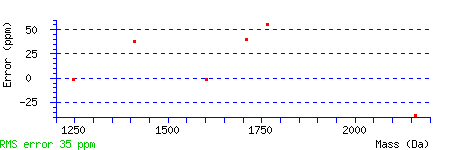


### Protein View

Match to: **K2C5_MOUSE** Score: **60** Expect: **0.011**

**(Q922U2) Keratin, type II cytoskeletal 5 (Cytokeratin-5) (CK-5) (Keratin-5) (K5)**

Nominal mass (M_r_): **61957**; Calculated pI value: **7.59**

NCBI BLAST search of [K2C5_MOUSE](http://www.ncbi.nlm.nih.gov/blast/Blast.cgi?ALIGNMENTS=50&ALIGNMENT_VIEW=Pairwise&AUTO_FORMAT=Semiauto&CDD_SEARCH=on&CLIENT=web&COMPOSITION_BASED_STATISTICS=on&DATABASE=nr&DESCRIPTIONS=100&ENTREZ_QUERY=(none)&EXPECT=10&FILTER=L&FORMAT_BLOCK_ON_RESPAGE=None&FORMAT_OBJECT=Alignment&FORMAT_TYPE=HTML&GAPCOSTS=11+1&I_THRESH=0.001&LAYOUT=TwoWindows&MATRIX_NAME=BLOSUM62&NCBI_GI=on&PAGE=Proteins&PROGRAM=blastp&QUERY=MSRQSSVSFRSGGSRSFSAASAITPSVSRTSFSSVSRSGGGGGGRISLGGACGAGGYGSRSLYNVGGSKRISYSSGGGSFRNQFGAGGFGFGGGAGSGFGFGGGAGSGFGFGGGAGFGGGYGGAGFPVCPPGGIQEVTVNQNLLTPLNLQIDPTIQRVRTEEREQIKTLNNKFASFIDKVRFLEQQNKVLDTKWALLQEQGTKTIKQNLDPLFEQYINNLRRQLDGVLGERGRLDSELRNMQDLVEDYKNKYEDEINKRTTAENEFVMLKKDVDAAYMNKVELEARVDALMDEINFMKMFFDAELSQMQTHVSDTSVVLSMDNNRSLDLDSIIAEVKAQYEDIANRSRTEAESWYQTKYEELQQTAGRHGDDLRNTKHEISEMNRMIQRLRSEIDNVKKQCANLQNAIAEAEQRGELALKDARNKLTELEEALQKAKQDMARLLREYQELMNTKLALDVEIATYRKLLEGEECRLSGEGVGPVNISVVTNSVSSGYGGGSSIGVGSGFGGGLGSGFAGGLGPRFTRGGGGLGLGSGLSVGGSGFSAGSSQGGMSFGSGGGSGSSVKFVSTTSSSRRSFKS&SERVICE=plain&SET_DEFAULTS.x=9&SET_DEFAULTS.y=5&SHOW_OVERVIEW=on&WORD_SIZE=3&END_OF_HTTPGET=Yes) against nr

Unformatted [sequence string](http://mascot/mascot/cgi/getseq.pl?Sprot+K2C5_MOUSE+seq) for pasting into other applications

Taxonomy: [Mus musculus](http://www.ncbi.nlm.nih.gov/htbin-post/Taxonomy/wgetorg?lvl=0&lin=f&id=10090)

Fixed modifications: Carbamidomethyl (C)

Variable modifications: Oxidation (M)

Cleavage by Trypsin: cuts C-term side of KR unless next residue is P

Number of mass values searched: **51**

Number of mass values matched: **11**

Sequence Coverage: **16%**

Matched peptides shown in **Bold Red**

**1** MSRQSSVSFR SGGSRSFSAA SAITPSVSR**T SFSSVSRSGG GGGGR**ISLGG

**51** ACGAGGYGSR SLYNVGGSKR ISYSSGGGSF RNQFGAGGFG FGGGAGSGFG

**101** FGGGAGSGFG FGGGAGFGGG YGGAGFPVCP PGGIQEVTVN QNLLTPLNLQ

**151** IDPTIQRVRT EEREQIKTLN NK**FASFIDKV RFLEQQNK**VL DTK**WALLQEQ**

**201 GTKTIK**QNLD PLFEQYINNL RRQLDGVLGE RGRLDSELRN MQDLVEDYKN

**251** KYEDEINK**RT TAENEFVMLK KDVDAAYMNK** VELEARVDAL MDEINFMKMF

**301** FDAELSQMQT HVSDTSVVLS MDNNRSLDLD SIIAEVKAQY EDIANR**SRTE**

**351 AESWYQTKYE ELQQTAGR**HG DDLRNTKHEI SEMNRMIQRL RSEIDNVKKQ

**401** CANLQNAIAE AEQRGELALK DARNKLTELE EALQKAKQDM ARLLREYQEL

**451** MNTKLALDVE IATYRKLLEG EECRLSGEGV GPVNISVVTN SVSSGYGGGS

**501** SIGVGSGFGG GLGSGFAGGL GPRFTRGGGG LGLGSGLSVG GSGFSAGSSQ

**551** GGMSFGSGGG SGSSVK**FVST TSSSR**RSFKS

  Residue Number  Increasing Mass  Decreasing Mass

**Start - End Observed Mr(expt) Mr(calc) Delta Miss Sequence**

**30 - 45 1455.6897 1454.6824 1454.6861 -0.0037 1 TSFSSVSRSGGGGGGR**

**173 - 181 1082.5985 1081.5912 1081.5920 -0.0008 1 FASFIDKVR**

**182 - 188 906.4639 905.4566 905.4606 -0.0040 0 FLEQQNK**

**194 - 206 1515.7370 1514.7297 1514.8456 -0.1159 1 WALLQEQGTKTIK**

**259 - 270 1438.7034 1437.6961 1437.7285 -0.0325 1 RTTAENEFVMLK**

**260 - 271 1410.7251 1409.7178 1409.7224 -0.0046 1 TTAENEFVMLKK**

**272 - 280 1026.5302 1025.5229 1025.4488 0.0741 0 DVDAAYMNK**

**347 - 358 1485.7264 1484.7191 1484.6895 0.0296 1 SRTEAESWYQTK**

**349 - 358 1242.5804 1241.5731 1241.5564 0.0167 0 TEAESWYQTK**

**359 - 368 1194.5923 1193.5850 1193.5676 0.0174 0 YEELQQTAGR**

**567 - 575 971.5018 970.4945 970.4719 0.0226 0 FVSTTSSSR**

**No match to:** 881.4036, 884.4098, 914.5031, 944.5182, 948.4951, 949.5183, 968.5043, 976.4866, 989.5134, 995.5228, 998.5067, 1012.5121, 1036.5403, 1062.5274, 1090.5412, 1093.5442, 1096.5465, 1107.5497, 1109.5066, 1111.5843, 1118.5298, 1128.5615, 1132.5294, 1144.5188, 1198.6949, 1203.6401, 1212.5876, 1214.6159, 1225.6243, 1234.6528, 1305.6218, 1357.7085, 1381.6748, 1599.8215, 1638.8493, 1712.9073, 1790.8877, 1794.8101, 2114.0076, 2873.4199


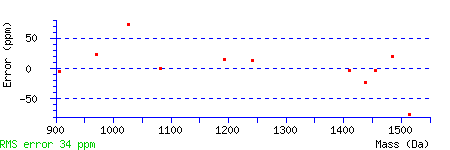


### Protein View

Match to: **TPH1_MOUSE** Score: **57** Expect: **0.021**

**(P17532) Tryptophan 5-hydroxylase 1 (EC 1.14.16.4) (Tryptophan 5-monooxygenase 1)**

Nominal mass (M_r_): **51881**; Calculated pI value: **6.06**

NCBI BLAST search of [TPH1_MOUSE](http://www.ncbi.nlm.nih.gov/blast/Blast.cgi?ALIGNMENTS=50&ALIGNMENT_VIEW=Pairwise&AUTO_FORMAT=Semiauto&CDD_SEARCH=on&CLIENT=web&COMPOSITION_BASED_STATISTICS=on&DATABASE=nr&DESCRIPTIONS=100&ENTREZ_QUERY=(none)&EXPECT=10&FILTER=L&FORMAT_BLOCK_ON_RESPAGE=None&FORMAT_OBJECT=Alignment&FORMAT_TYPE=HTML&GAPCOSTS=11+1&I_THRESH=0.001&LAYOUT=TwoWindows&MATRIX_NAME=BLOSUM62&NCBI_GI=on&PAGE=Proteins&PROGRAM=blastp&QUERY=MIEDNKENKENKDHSSERGRVTLIFSLENEVGGLIKVLKIFQENHVSLLHIESRKSKQRNSEFEIFVDCDISREQLNDIFPLLKSHATVLSVDSPDQLTAKEDVMETVPWFPKKISDLDFCANRVLLYGSELDADHPGFKDNVYRRRRKYFAELAMNYKHGDPIPKIEFTEEEIKTWGTIFRELNKLYPTHACREYLRNLPLLSKYCGYREDNIPQLEDVSNFLKERTGFSIRPVAGYLSPRDFLSGLAFRVFHCTQYVRHSSDPLYTPEPDTCHELLGHVPLLAEPSFAQFSQEIGLASLGASEETVQKLATCYFFTVEFGLCKQDGQLRVFGAGLLSSISELKHALSGHAKVKPFDPKIACKQECLITSFQDVYFVSESFEDAKEKMREFAKTVKRPFGLKYNPYTQSVQVLRDTKSITSAMNELRYDLDVISDALARVTRWPSV&SERVICE=plain&SET_DEFAULTS.x=9&SET_DEFAULTS.y=5&SHOW_OVERVIEW=on&WORD_SIZE=3&END_OF_HTTPGET=Yes) against nr

Unformatted [sequence string](http://mascot/mascot/cgi/getseq.pl?Sprot+TPH1_MOUSE+seq) for pasting into other applications

Taxonomy: [Mus musculus](http://www.ncbi.nlm.nih.gov/htbin-post/Taxonomy/wgetorg?lvl=0&lin=f&id=10090)

Fixed modifications: Carbamidomethyl (C)

Variable modifications: Oxidation (M)

Cleavage by Trypsin: cuts C-term side of KR unless next residue is P

Number of mass values searched: **30**

Number of mass values matched: **7**

Sequence Coverage: **17%**

Matched peptides shown in **Bold Red**

**1** MIEDNKENKE NKDHSSERGR VTLIFSLENE VGGLIKVLK**I FQENHVSLLH**

**51 IESRK**SKQRN SEFEIFVDCD ISREQLNDIF PLLKSHATVL SVDSPDQLTA

**101** K**EDVMETVPW FPKK**ISDLDF CANRVLLYGS ELDADHPGFK DNVYRRRRK**Y**

**151 FAELAMNYKH GDPIPK**IEFT EEEIKTWGTI FRELNKLYPT HACREYLRNL

**201** PLLSKYCGYR EDNIPQLEDV SNFLKERTGF SIRPVAGYLS PR**DFLSGLAF**

**251 RVFHCTQYVR** HSSDPLYTPE PDTCHELLGH VPLLAEPSFA QFSQEIGLAS

**301** LGASEETVQK LATCYFFTVE FGLCKQDGQL RVFGAGLLSS ISELKHALSG

**351** HAKVKPFDPK IACKQECLIT SFQDVYFVSE SFEDAKEKMR EFAKTVKRPF

**401** GLK**YNPYTQS VQVLRDTK**SI TSAMNELRYD LDVISDALAR VTRWPSV

  Residue Number  Increasing Mass  Decreasing Mass

**Start - End Observed Mr(expt) Mr(calc) Delta Miss Sequence**

**40 - 55 1950.0460 1949.0387 1949.0482 -0.0095 1 IFQENHVSLLHIESRK**

**102 - 114 1621.7090 1620.7017 1620.7857 -0.0840 1 EDVMETVPWFPKK**  Oxidation (M)

**150 - 166 1994.0890 1993.0817 1992.9767 0.1050 1 YFAELAMNYKHGDPIPK**

**243 - 260 2216.1150 2215.1077 2215.0996 0.0081 1 DFLSGLAFRVFHCTQYVR**

**252 - 260 1209.5980 1208.5907 1208.5760 0.0147 0 VFHCTQYVR**

**404 - 415 1467.7950 1466.7877 1466.7517 0.0360 0 YNPYTQSVQVLR**

**404 - 418 1811.9020 1810.8947 1810.9213 -0.0266 1 YNPYTQSVQVLRDTK**

**No match to:** 870.4490, 1322.7490, 1363.7420, 1381.7400, 1398.7690, 1411.7530, 1536.8960, 1619.7410, 1635.7180, 1695.8630, 1778.9820, 1914.8290, 1960.0820, 1976.0780, 2005.0750, 2087.0600, 2207.1010, 2307.2440, 2323.2460, 2793.4110, 2817.3870, 3171.6900, 3714.8390


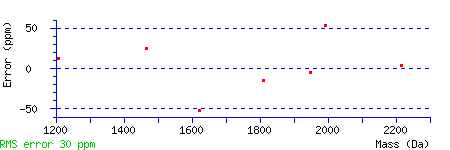


### Protein View

Match to: **CT160_MOUSE** Score: **55** Expect: **0.033**

**(Q8VCC6) Protein C20orf160 homolog**

Nominal mass (M_r_): **22060**; Calculated pI value: **9.51**

NCBI BLAST search of [CT160_MOUSE](http://www.ncbi.nlm.nih.gov/blast/Blast.cgi?ALIGNMENTS=50&ALIGNMENT_VIEW=Pairwise&AUTO_FORMAT=Semiauto&CDD_SEARCH=on&CLIENT=web&COMPOSITION_BASED_STATISTICS=on&DATABASE=nr&DESCRIPTIONS=100&ENTREZ_QUERY=(none)&EXPECT=10&FILTER=L&FORMAT_BLOCK_ON_RESPAGE=None&FORMAT_OBJECT=Alignment&FORMAT_TYPE=HTML&GAPCOSTS=11+1&I_THRESH=0.001&LAYOUT=TwoWindows&MATRIX_NAME=BLOSUM62&NCBI_GI=on&PAGE=Proteins&PROGRAM=blastp&QUERY=MEYEAKKGKKGFVSPIRRLVFPKAARQAAFRSSVSRRPLHSMPLYPPDYLIDPHILLCDYLEKEVKFLGHLTWVTSSLNPSSRDELLQLLDTARQLKELPLKTTPEQDSILSLSARCLLLTWRDNEELILRIPTHEIAAASYLQDDALHLLVLKTGAARVWGWTLCLRAWMAAREVRVAIPALRAPRPRNDG&SERVICE=plain&SET_DEFAULTS.x=9&SET_DEFAULTS.y=5&SHOW_OVERVIEW=on&WORD_SIZE=3&END_OF_HTTPGET=Yes) against nr

Unformatted [sequence string](http://mascot/mascot/cgi/getseq.pl?Sprot+CT160_MOUSE+seq) for pasting into other applications

Taxonomy: [Mus musculus](http://www.ncbi.nlm.nih.gov/htbin-post/Taxonomy/wgetorg?lvl=0&lin=f&id=10090)

Fixed modifications: Carbamidomethyl (C)

Variable modifications: Oxidation (M)

Cleavage by Trypsin: cuts C-term side of KR unless next residue is P

Number of mass values searched: **16**

Number of mass values matched: **5**

Sequence Coverage: **20%**

Matched peptides shown in **Bold Red**

**1** **MEYEAKK**GKK GFVSPIRRLV FPKAAR**QAAF RSSVSR**RPLH SMPLYPPDYL

**51** IDPHILLCDY LEKEVKFLGH LTWVTSSLNP SSRDELLQLL DTARQLKELP

**101** LKTTPEQDSI LSLSAR**CLLL TWRDNEELIL R**IPTHEIAAA SYLQDDALHL

**151** LVLKTGAARV WGWTLCLRAW MAAREVRVAI PALR**APRPRN DG**

  Residue Number  Increasing Mass  Decreasing Mass

**Start - End Observed Mr(expt) Mr(calc) Delta Miss Sequence**

**1 - 7 914.4709 913.4636 913.4215 0.0421 1 MEYEAKK**  Oxidation (M)

**27 - 36 1108.6221 1107.6148 1107.5784 0.0364 1 QAAFRSSVSR**

**117 - 123 961.5574 960.5502 960.5215 0.0287 0 CLLLTWR**

**124 - 131 1001.5703 1000.5631 1000.5189 0.0442 0 DNEELILR**

**185 - 192 882.4413 881.4340 881.4467 -0.0127 1 APRPRNDG**

**No match to:** 948.5020, 949.5157, 960.4859, 1060.0516, 1066.0654, 1114.5122, 1214.6713, 1305.6376, 1357.7690, 1363.7666, 2212.2082


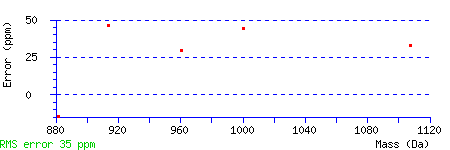


### Protein View

Match to: **DYN2_MOUSE** Score: **68** Expect: **0.0069**

**(P39054) Dynamin-2 (EC 3.6.5.5) (Dynamin UDNM)**

Nominal mass (M_r_): **98426**; Calculated pI value: **7.02**

NCBI BLAST search of [DYN2_MOUSE](http://www.ncbi.nlm.nih.gov/blast/Blast.cgi?ALIGNMENTS=50&ALIGNMENT_VIEW=Pairwise&AUTO_FORMAT=Semiauto&CDD_SEARCH=on&CLIENT=web&COMPOSITION_BASED_STATISTICS=on&DATABASE=nr&DESCRIPTIONS=100&ENTREZ_QUERY=(none)&EXPECT=10&FILTER=L&FORMAT_BLOCK_ON_RESPAGE=None&FORMAT_OBJECT=Alignment&FORMAT_TYPE=HTML&GAPCOSTS=11+1&I_THRESH=0.001&LAYOUT=TwoWindows&MATRIX_NAME=BLOSUM62&NCBI_GI=on&PAGE=Proteins&PROGRAM=blastp&QUERY=MGNRGMEELIPLVNKLQDAFSSIGQSCHLDLPQIAVVGGQSAGKSSVLENFVGRDFLPRGSGIVTRRPLILQLIFSKTEYAEFLHCKSKKFTDFDEVRQEIEAETDRVTGTNKGISPVPINLRVYSPHVLNLTLIDLPGITKVPVGDQPPDIEYQIKDMILQFISRESSLILAVTPANMDLANSDALKLAKEVDPQGLRTIGVITKLDLMDEGTDARDVLENKLLPLRRGYIGVVNRSQKDIEGKKDIRAALAAERKFFLSHPAYRHMADRMGTPHLQKTLNQQLTNHIRESLPTLRSKLQSQLLSLEKEVEEYKNFRPDDPTRKTKALLQMVQQFGVDFEKRIEGSGDQVDTLELSGGARINRIFHERFPFELVKMEFDEKDLRREISYAIKNIHGVRTGLFTPDLAFEAIVKKQVVKLKEPCLKCVDLVIQELISTVRQCTSKLSSYPRLREETERIVTTYIREREGRTKDQILLLIDIEQSYINTNHEDFIGFANAQQRSTQLNKKRAIPNQGEILVIRRGWLTINNISLMKGGSKEYWFVLTAESLSWYKDEEEKEKKYMLPLDNLKIRDVEKGFMSNKHVFAIFNTEQRNVYKDLRQIELACDSQEDVDSWKASFLRAGVYPEKDQAENEDGAQENTFSMDPQLERQVETIRNLVDSYVAIINKSIRDLMPKTIMHLMINNTKAFIHHELLAYLYSSADQSSLMEESAEQAQRRDDMLRMYHALKEALNIIGDISTSTVSTPVPPPVDDTWLQNTSGHSPTPQRRPVSSVHPPGRPPAVRGPTPGPPLIPMPVGATSSFSAPPIPSRPGPQSVFANNDPFSAPPQIPSRPARIPPGIPPGVPSRRAPAAPSRPTIIRPAEPSLLD&SERVICE=plain&SET_DEFAULTS.x=9&SET_DEFAULTS.y=5&SHOW_OVERVIEW=on&WORD_SIZE=3&END_OF_HTTPGET=Yes) against nr

Unformatted [sequence string](http://mascot/mascot/cgi/getseq.pl?Sprot+DYN2_MOUSE+seq) for pasting into other applications

Taxonomy: [Mus musculus](http://www.ncbi.nlm.nih.gov/htbin-post/Taxonomy/wgetorg?lvl=0&lin=f&id=10090)

Fixed modifications: Carbamidomethyl (C)

Variable modifications: Oxidation (M)

Cleavage by Trypsin: cuts C-term side of KR unless next residue is P

Number of mass values searched: **20**

Number of mass values matched: **9**

Sequence Coverage: **12%**

Matched peptides shown in **Bold Red**

**1** MGNR**GMEELI PLVNK**LQDAF SSIGQSCHLD LPQIAVVGGQ SAGKSSVLEN

**51** FVGRDFLPRG SGIVTRRPLI LQLIFSK**TEY AEFLHCK**SKK FTDFDEVRQE

**101** IEAETDRVTG TNKGISPVPI NLRVYSPHVL NLTLIDLPGI TKVPVGDQPP

**151** DIEYQIKDMI LQFISRESSL ILAVTPANMD LANSDALK**LA KEVDPQGLRT**

**201 IGVITK**LDLM DEGTDARDVL ENKLLPLRRG YIGVVNRSQK DIEGKKDIRA

**251** ALAAERKFFL SHPAYR**HMAD RMGTPHLQK**T LNQQLTNHIR ESLPTLRSKL

**301** QSQLLSLEKE VEEYKNFRPD DPTRK**TKALL QMVQQFGVDF EKRIEGSGDQ**

**351 VDTLELSGGA R**INRIFHERF PFELVKMEFD EKDLRREISY AIKNIHGVRT

**401** GLFTPDLAFE AIVKKQVVKL KEPCLKCVDL VIQELISTVR QCTSKLSSYP

**451** RLREETERIV TTYIREREGR TKDQILLLID IEQSYINTNH EDFIGFANAQ

**501** QRSTQLNKKR AIPNQGEILV IRR**GWLTINN ISLMK**GGSKE YWFVLTAESL

**551** SWYKDEEEKE KKYMLPLDNL KIRDVEKGFM SNK**HVFAIFN TEQR**NVYKDL

**601** RQIELACDSQ EDVDSWKASF LRAGVYPEKD QAENEDGAQE NTFSMDPQLE

**651** RQVETIRNLV DSYVAIINKS IRDLMPKTIM HLMINNTKAF IHHELLAYLY

**701** SSADQSSLME ESAEQAQRRD DMLRMYHALK EALNIIGDIS TSTVSTPVPP

**751** PVDDTWLQNT SGHSPTPQRR PVSSVHPPGR PPAVRGPTPG PPLIPMPVGA

**801** TSSFSAPPIP SRPGPQSVFA NNDPFSAPPQ IPSRPARIPP GIPPGVPSRR

**851** APAAPSRPTI IRPAEPSLLD

  Residue Number  Increasing Mass  Decreasing Mass

**Start - End Observed Mr(expt) Mr(calc) Delta Miss Sequence**

**5 - 15 1258.6860 1257.6787 1257.6638 0.0149 0 GMEELIPLVNK**  Oxidation (M)

**78 - 87 1297.6740 1296.6667 1296.5808 0.0859 0 TEYAEFLHCK**

**189 - 199 1225.6800 1224.6727 1224.6826 -0.0098 1 LAKEVDPQGLR**

**192 - 206 1625.9270 1624.9197 1624.9147 0.0050 1 EVDPQGLRTIGVITK**

**267 - 279 1537.8830 1536.8757 1536.7289 0.1468 1 HMADRMGTPHLQK**  Oxidation (M)

**326 - 342 1981.9340 1980.9267 1981.0342 -0.1075 1 TKALLQMVQQFGVDFEK**

**343 - 361 1960.0520 1959.0447 1958.9657 0.0791 1 RIEGSGDQVDTLELSGGAR**

**524 - 535 1405.8100 1404.8027 1404.7435 0.0593 0 GWLTINNISLMK**  Oxidation (M)

**584 - 594 1361.7780 1360.7707 1360.6887 0.0820 0 HVFAIFNTEQR**

**No match to:** 870.4378, 1004.5600, 1061.5650, 1237.5750, 1449.8250, 1455.8100, 1468.7830, 1493.8570, 1581.9050, 1713.9930, 2212.1390


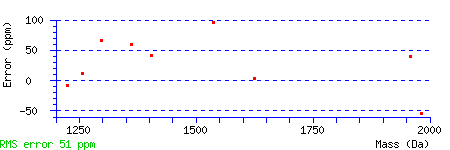


### Protein View

Match to: **KPYM_MOUSE** Score: **53** Expect: **0.05**

**(P52480) Pyruvate kinase, isozyme M2 (EC 2.7.1.40)**

Nominal mass (M_r_): **58289**; Calculated pI value: **7.42**

NCBI BLAST search of [KPYM_MOUSE](http://www.ncbi.nlm.nih.gov/blast/Blast.cgi?ALIGNMENTS=50&ALIGNMENT_VIEW=Pairwise&AUTO_FORMAT=Semiauto&CDD_SEARCH=on&CLIENT=web&COMPOSITION_BASED_STATISTICS=on&DATABASE=nr&DESCRIPTIONS=100&ENTREZ_QUERY=(none)&EXPECT=10&FILTER=L&FORMAT_BLOCK_ON_RESPAGE=None&FORMAT_OBJECT=Alignment&FORMAT_TYPE=HTML&GAPCOSTS=11+1&I_THRESH=0.001&LAYOUT=TwoWindows&MATRIX_NAME=BLOSUM62&NCBI_GI=on&PAGE=Proteins&PROGRAM=blastp&QUERY=PKPHSEAGTAFIQTQQLHAAMADTFLEHMCRLDIDSAPITARNTGIICTIGPASRSVEMLKEMIKSGMNVARLNFSHGTHEYHAETIKNVREATESFASDPILYRPVAVALDTKGPEIRTGLIKGSGTAEVELKKGATLKITLDNAYMEKCDENILWLDYKNICKVVEVGSKIYVDDGLISLQVKEKGADFLVTEVENGGSLGSKKGVNLPGAAVDLPAVSEKDIQDLKFGVEQDVDMVFASFIRKAADVHEVRKVLGEKGKNIKIISKIENHEGVRRFDEILEASDGIMVARGDLGIEIPAEKVFLAQKMMIGRCNRAGKPVICSTQMLEIMIKKPRPTRAEGSDVANAVLDGADCIMLSGETAKGDYPLEAVRMQHLIAREAEAAIYHLQLFEELRRLAPITSDPTEAAAVGAVEASFKCCSGAIIVLTKSGRSAHQVARYRPRAPIIAVTRNPQTARQAHLYRGIFPVLCKDAVLNAWAEDVDLRVNLAMDVGKARGFFKKGDVVIVLTGWRPGSGFTNTMRVVPVP&SERVICE=plain&SET_DEFAULTS.x=9&SET_DEFAULTS.y=5&SHOW_OVERVIEW=on&WORD_SIZE=3&END_OF_HTTPGET=Yes) against nr

Unformatted [sequence string](http://mascot/mascot/cgi/getseq.pl?Sprot+KPYM_MOUSE+seq) for pasting into other applications

Taxonomy: [Mus musculus](http://www.ncbi.nlm.nih.gov/htbin-post/Taxonomy/wgetorg?lvl=0&lin=f&id=10090)

Fixed modifications: Carbamidomethyl (C)

Variable modifications: Oxidation (M)

Cleavage by Trypsin: cuts C-term side of KR unless next residue is P

Number of mass values searched: **80**

Number of mass values matched: **12**

Sequence Coverage: **30%**

Matched peptides shown in **Bold Red**

**1** PKPHSEAGTA FIQTQQLHAA MADTFLEHMC RLDIDSAPIT AR**NTGIICTI**

**51 GPASR**SVEML KEMIKSGMNV ARLNFSHGTH EYHAETIKNV REATESFASD

**101** PILYRPVAVA LDTKGPEIRT GLIKGSGTAE VELKKGATLK **ITLDNAYMEK**

**151 CDENILWLDY K**NICKVVEVG SKIYVDDGLI SLQVKEKGAD FLVTEVENGG

**201** SLGSKKGVNL PGAAVDLPAV SEKDIQDLKF GVEQDVDMVF ASFIRKAADV

**251** HEVRKVLGEK GKNIKIISK**I ENHEGVRRFD EILEASDGIM VARGDLGIEI**

**301 PAEK**VFLAQK **MMIGRCNRAG KPVICSTQML EIMIK**KPRPT R**AEGSDVANA**

**351 VLDGADCIML SGETAK**GDYP LEAVRMQHLI AREAEAAIYH LQLFEELRRL

**401** APITSDPTEA AAVGAVEASF KCCSGAIIVL TKSGR**SAHQV ARYRPR**APII

**451** AVTRNPQTAR QAHLYR**GIFP VLCKDAVLNA WAEDVDLRVN LAMDVGKAR**G

**501** FFKKGDVVIV LTGWRPGSGF TNTMRVVPVP

  Residue Number  Increasing Mass  Decreasing Mass

**Start - End Observed Mr(expt) Mr(calc) Delta Miss Sequence**

**43 - 55 1359.7026 1358.6953 1358.6976 -0.0023 0 NTGIICTIGPASR**

**141 - 161 2663.2808 2662.2735 2662.2294 0.0441 1 ITLDNAYMEKCDENILWLDYK**  Oxidation (M)

**270 - 278 1109.5139 1108.5067 1108.5737 -0.0670 1 IENHEGVRR**

**278 - 293 1837.9420 1836.9347 1836.9039 0.0308 1 RFDEILEASDGIMVAR**  Oxidation (M)

**279 - 304 2788.1603 2787.1530 2787.3999 -0.2470 1 FDEILEASDGIMVARGDLGIEIPAEK**

**311 - 318 1037.5142 1036.5069 1036.4728 0.0341 1 MMIGRCNR**

**311 - 318 1053.5105 1052.5033 1052.4677 0.0355 1 MMIGRCNR**  Oxidation (M)

**316 - 335 2349.2090 2348.2017 2348.1836 0.0181 1 CNRAGKPVICSTQMLEIMIK**

**342 - 366 2510.1488 2509.1416 2509.1311 0.0104 0 AEGSDVANAVLDGADCIMLSGETAK**  Oxidation (M)

**436 - 446 1340.6773 1339.6700 1339.7221 -0.0521 1 SAHQVARYRPR**

**467 - 488 2501.2555 2500.2482 2500.2783 -0.0301 1 GIFPVLCKDAVLNAWAEDVDLR**

**489 - 499 1189.5690 1188.5617 1188.6284 -0.0667 1 VNLAMDVGKAR**  Oxidation (M)
